# From Folding Mechanics to Robotic Function: A Unified Modeling Framework for Compliant Origami

**DOI:** 10.1002/advs.76715

**Published:** 2026-07-24

**Authors:** Bohan Zhang, Bo Wang, Huajiang Ouyang, Zhigang Wu, Haohao Bi, Jiawei Xu, Mingchao Liu, Weicheng Huang

**Affiliations:** ^1^ Department of Engineering Mechanics Northwestern Polytechnical University Xi'an China; ^2^ Shenzhen Research Institute of Northwestern Polytechnical University Shenzhen China; ^3^ School of Mechanical Engineering Southwest Jiaotong University Chengdu China; ^4^ School of Engineering University of Liverpool Liverpool UK; ^5^ School of Aeronautics and Astronautics Sun Yat‐sen University Shenzhen Guangdong China; ^6^ School of Science Qingdao University of Technology Qingdao China; ^7^ Department of Mechanical Engineering University of Birmingham Birmingham UK; ^8^ School of Engineering Newcastle University Newcastle upon Tyne UK

**Keywords:** computational modeling, discrete differential geometry, mechanical compliance, nonlinear mechanics, origami robots

## Abstract

Origami‐inspired architectures offer a powerful route toward lightweight, reconfigurable, and programmable robotic systems. Yet, a unified mechanics framework capable of seamlessly bridging rigid folding, elastic deformation, and stability‐driven transitions in compliant origami remains lacking. Here, we introduce a geometry‐consistent modeling framework based on discrete differential geometry (DDG) that unifies panel elasticity and crease rotation within a single variational formulation. By embedding crease–panel coupling directly into a mid‐edge geometric discretization, the framework naturally captures rigid‐folding limits, distributed bending, multistability, and nonlinear dynamic snap‐through within one mechanically consistent structure. This unified description enables programmable control of stability and deformation across rigid and compliant regimes, allowing origami structures to transition from static folding mechanisms to active robotic modules. An implicit dynamic formulation incorporating gravity, contact, friction, and magnetic actuation further supports strongly coupled multiphysics simulations. Through representative examples spanning single‐fold bifurcation, deployable Miura membranes, bistable Waterbomb modules, and Kresling‐based crawling robots, we demonstrate how geometry‐driven mechanics directly informs robotic functionality. This work establishes discrete differential geometry as a foundational design language for intelligent origami robotics, enabling predictive modeling, stability programming, and mechanics‐guided robotic actuation within a unified computational platform.

## Graphical abstract

A geometry‐consistent DDG framework captures compliant origami deformation from rigid‐like folding to soft panel bending. By tuning crease and panel stiffness, bistable snap‐through responses transform into smooth monostable deformation, enabling programmable stability and robot‐scale locomotion. This mechanics‐guided model links origami structure, nonlinear response, and robotic function in a unified simulation framework.

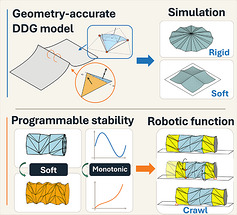

## 1 Introduction

Origami‐inspired architectures have emerged as a powerful paradigm for designing lightweight, compact, and highly reconfigurable robotic systems [1, 2]. By encoding large shape transformations into geometric folding patterns, origami structures enable deployable space systems [3], adaptive mechanical metamaterials [4, 5], robotic actuators [6], and bio‐inspired locomotion platforms [7]. In recent years, the integration of origami principles with soft robotics has enabled robots capable of rapid deployment, multistable actuation, and programmable morphing, offering new opportunities for intelligent machines that exploit structural mechanics as a functional design element [8, 9, 10]. Despite these advances, predicting and controlling the mechanics of compliant origami systems remains challenging due to the strong coupling between folding kinematics, panel elasticity, and nonlinear stability transitions [11].

Existing modeling strategies for origami structures can be broadly categorized into several paradigms according to their underlying mechanical assumptions [12]. Rigid‐panel models represent origami as assemblies of rigid facets connected by rotational hinges, and efficiently capture folding compatibility, deployment paths, and degrees of freedom in rigid origami [13, 14]. However, they neglect distributed bending and in‐plane deformation of panels, which limits their applicability to compliant origami and soft robotic systems where elasticity is essential. Bar‐and‐hinge models approximate origami surfaces using skeletal networks, where bars describe in‐plane stretching and rotational springs account for folding or bending resistance [15, 16]. Although computationally efficient, their mechanical response often depends on heuristic stiffness assignments and lacks a direct geometric description of the compliant panels. Continuum‐based approaches, most commonly finite element methods (FEM), provide a general framework for thin structures undergoing large deformation and can capture both stretching and bending of origami panels [17, 18, 19]. Previous dynamic studies on rigid‐foldable origami, including stacked Miura–ori bistability and Kresling patterns, have demonstrated the effectiveness of reduced rigid‐folding models in analyzing motion generation, stability switching, and dynamic actuation characteristics relevant to origami robotic systems [20, 21]. However, extending such transient analyzes to flexible origami structures typically entails substantially higher computational cost [20]. Consequently, bridging rigid folding kinematics with distributed shell deformation within a unified and geometrically consistent framework remains an outstanding challenge.

Beyond prescribed folding motion, compliant origami structures can exploit mechanical stability as a programmable design variable. For a given origami geometry, variations in physical parameters such as crease stiffness and material compliance can reshape the energy landscape, thereby enabling tunable mechanical behaviors [22], including bistability, multistability, snap‐through transitions, and deformation‐mode selection [23, 24, 25]. This programmability provides a mechanics‐guided route for robotic functions, including actuation, locomotion, gripping, and adaptive shape transformation [1, 26, 27]. However, resolving such behavior requires a model that can simultaneously account for folding compatibility, crease rotations, panel deformation, and nonlinear dynamics. In particular, the framework should remain efficient in the near‐rigid regime while providing sufficient accuracy when compliant panels undergo distributed stretching, bending, twisting, and crease–panel coupling.

Discrete differential geometry (DDG), which directly discretizes geometric invariants including metric and curvature, provides a natural foundation for linking surface geometry with mechanical response in thin structures [28, 29]. This feature is particularly suitable for compliant origami, where folding motion, panel deformation, and crease rotation are intrinsically governed by changes in geometry. By preserving intrinsic geometric relationships, DDG‐based formulations can robustly handle large rotations and deformations [30]. This work develops a DDG‐based framework for the mechanics‐guided design of compliant origami robots. Panels and creases are represented within a common geometric discretization, enabling a consistent description of shell deformation and fold rotation.

In this work, we develop a robot‐level DDG simulation framework for the mechanics‐guided design of compliant origami robotic systems. Beyond reproducing prescribed folding motions, the framework introduces programmable stability as a design concept, showing how crease–panel stiffness competition reshapes the energy landscape and regulates deformation modes, stability transitions, and actuation responses. Through representative origami systems [8, 31, 32], we demonstrate the potential of the same formulation to bridge rigid‐like folding and compliant deformation regimes. The analysis further extends these structural and mechanical concepts toward robotic design, where stiffness‐dependent mechanical responses are linked to functional behaviors such as shape transformation, actuation, and locomotion. By connecting geometric mechanics, programmable stability, and robot‐scale dynamics, this work provides a mechanics‐guided computational foundation for the design of compliant origami robotic systems.

## 2 A Geometry‐Consistent Discrete Mechanics Framework for Compliant Origami

This section presents a geometry‐consistent discrete mechanics framework for compliant origami robots. Rather than treating folding kinematics and elastic deformation separately, the formulation describes panel deformation and crease mechanics within a unified variational setting.

Denoting the generalized coordinates by q, the total elastic potential energy is defined as

(1) $$\Pi \left(q\right)=U_{\mathrm{shell}}\left(q\right)+U_{\mathrm{crease}}\left(q\right),$$

where $U_{\mathrm{shell}}$ accounts for energies of the deformable panels, discretized using a discrete differential geometry (DDG) formulation [28], and $U_{\mathrm{crease}}$ describes localized rotational stiffness along fold lines. This decomposition enables rigid‐like folding and compliant panel deformation to be captured within the same mechanical framework.

To include transient dynamics, the system is described by the Lagrangian

(2) $$L\left(q,\dot{q}\right)=T\left(\dot{q}\right)-\Pi \left(q\right),$$

where $\dot{q}$ denotes the generalized velocity and T is the kinetic energy. This unified variational formulation captures rigid‐like folding, compliant deformation, and their dynamic coupling, thereby enabling the simulation of nonlinear dynamics [33], multistability [34, 35], snap‐through transitions [36], frictional contact [37], and multi‐physics coupling [38]. The shell and crease energy terms are introduced below, with detailed derivations provided in the Supporting Information.

### 2.1 Equation of Motion and Generalized Degrees of Freedom

For realistic modeling of compliant origami robots, the formulation must account for inertia, damping, environmental interactions, and field‐driven actuation in addition to elastic deformation. These contributions are assembled in the generalized coordinate space of the origami system. Taking the variation of the Lagrangian in Equation (2) and incorporating non‐conservative forces, the nonlinear equation of motion is written as

(3) $$M\ddot{q}\left(t\right)+C\dot{q}\left(t\right)+f_{\mathrm{int}}\left(q\right)=f_{\mathrm{ext}}\left(t\right),$$

where M is the mass matrix derived from the kinetic energy, C is the damping matrix, $f_{\mathrm{int}}=\partial \Pi /\partial q$ is the internal elastic force, and $f_{\mathrm{ext}}$ collects external and environmental forces, including gravity, contact, friction, and magnetic actuation.

**FIGURE 1:**
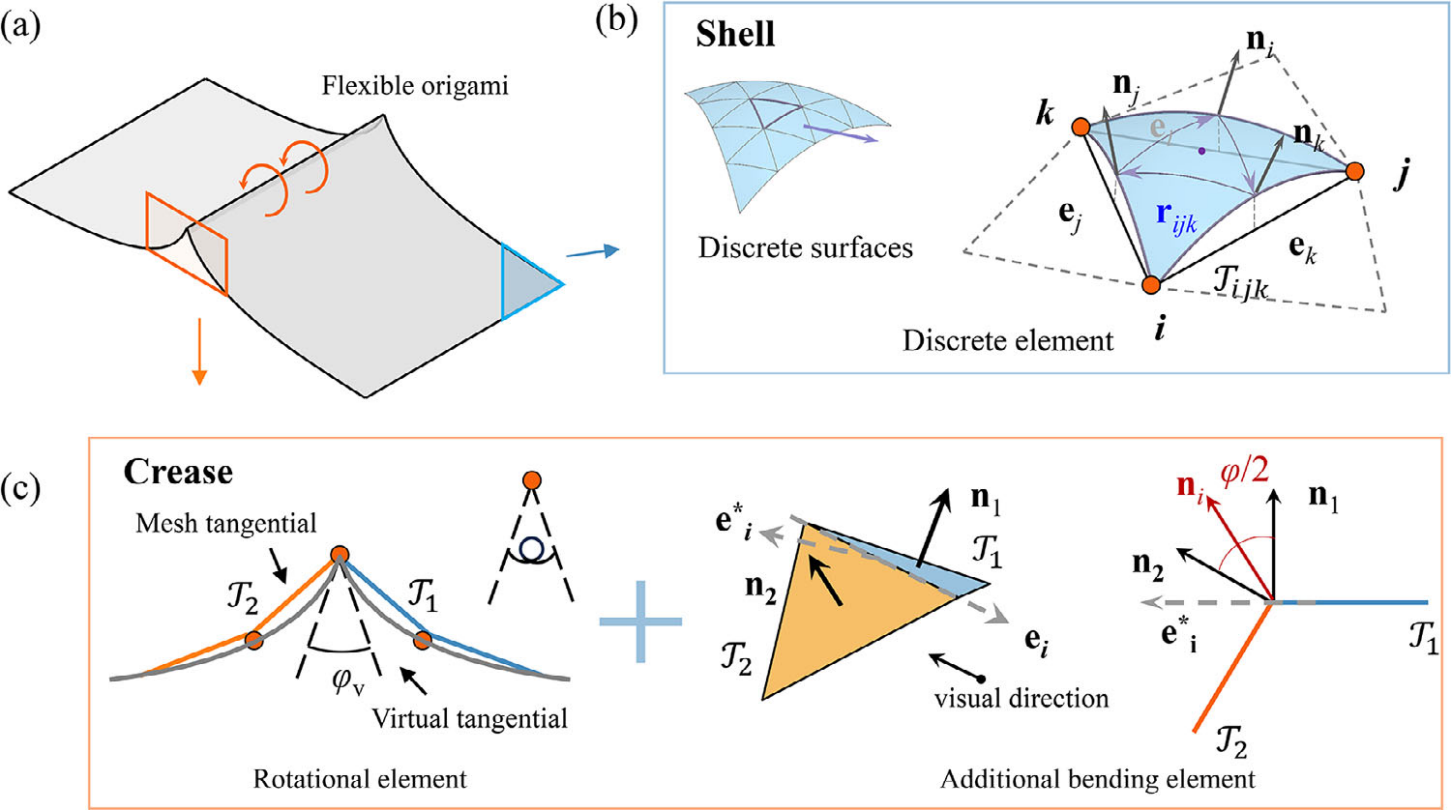
Schematic illustration of a flexible origami structure and its discrete modeling framework. (a) A minimal origami unit consists of a crease (highlighted in orange) connecting two deformable shell panels (highlighted in blue). (b) The shell module is discretized by triangular elements and described using DDG‐based metric and curvature measures. (c)The crease module is represented by rotational and bending elements.

As illustrated in Figure 1a compliant origami structure is represented by two coupled modules: deformable shell panels and crease elements. The generalized coordinates therefore consist of two sets of variables. The first set contains the spatial positions of all shell vertices,

$$v_{\left(a\right)}\left(t\right)=\left[\begin{array}{c} x_{\left(a\right)}\left(t\right)\;y_{\left(a\right)}\left(t\right)\;z_{\left(a\right)}\left(t\right) \end{array}\right],\;a=1,\text{…},N_{1},$$

where $N_{1}$ is the total number of vertices. The second set contains the virtual folding angles associated with the crease elements,

$$\varphi_{v,(b)}\left(t\right),\;b=1,\text{…},N_{2},$$

where $N_{2}$ is the total number of creases. The generalized coordinate vector is therefore written as

(4) $$q\left(t\right)={\left[v_{\left(1\right)}\left(t\right),\cdots ,v_{\left(N_{1}\right)}\left(t\right),\varphi_{v,(1)}\left(t\right),\cdots ,\varphi_{v,(N_{2})}\left(t\right)\right]}^{T},$$

which gives a total of $3N_{1}+N_{2}$ degrees of freedom.

The resulting nonlinear system is integrated using an implicit Euler scheme and solved by Newton iterations, with the tangent operator obtained from the derivative of the internal force, i.e., $\partial f_{\mathrm{int}}/\partial q=\partial^{2}\Pi /\partial q^{2}$, and updated according to the instantaneous configuration.

Equation (3) provides a common dynamic description for rigid‐like folding, compliant panel deformation, stability transitions, and robot‐scale interactions with the environment. The construction of the shell and crease energies is introduced in the following subsections, while the detailed numerical implementation is provided in the Section S1.2, S1.3. The source code of the simulation framework is publicly available in an open‐access repository [39].

### 2.2 Discrete Elastic Energy of Compliant Panels

The deformable panels of the origami robot are modeled as thin shells and discretized by triangular meshes, as illustrated in Figure 1b. In the present framework, each crease is treated as a free boundary of the adjacent panels, while mechanical coupling between panels is introduced through dedicated crease elements. This treatment allows panel deformation and crease rotation to be modeled separately but coupled within the same variational formulation.

To describe large deformation of the panels, we adopt a discrete differential geometry (DDG) shell formulation [28, 29, 40, 41, 42]. The discrete first and second fundamental forms are used to characterize in‐plane metric distortion and out‐of‐plane curvature variation, respectively. Compared with displacement‐based small‐strain descriptions, these geometric measures provide a rotation‐invariant description of panel stretching and bending, which is essential for compliant origami undergoing large rotations.

For each triangular element $T_{ijk}$, the membrane and bending strains are defined from the changes in the discrete metric and curvature relative to the reference configuration,

(5) $$\varepsilon_{ijk}={\bar{A}}_{ijk}^{-1}A_{ijk}-I,\;\kappa_{ijk}={\bar{A}}_{ijk}^{-1}\left(B_{ijk}-{\bar{B}}_{ijk}\right),$$

where $A_{ijk}$ and $B_{ijk}$ are the discrete first and second fundamental forms, and the overbar denotes the reference configuration.

The shell energy is written as the sum of stretching and bending contributions,

(6) $$U_{\mathrm{shell}}=\sum_{ijk}^{N_{t}}\left[\frac{h}{8}{\left\|\varepsilon_{ijk}\right\|}_{SV}^{2}+\frac{h^{3}}{24}{\left\|\kappa_{ijk}\right\|}_{SV}^{2}\right]\sqrt{\det({\bar{A}}_{ijk})},$$

where h is the shell thickness, $N_{t}$ is the number of triangular elements, and ${∥\cdot ∥}_{SV}^{2}$ denotes the St. Venant–Kirchhoff elastic energy density [43]. The explicit construction of the discrete fundamental forms and the constitutive expression are provided in the Section S1.2.

### 2.3 Geometry‐Consistent Modeling of Crease

We next introduce the discrete treatment of creases in compliant origami. In the present framework, two independent shell panels are connected by a series of crease elements distributed along the prescribed fold line. Each crease element is geometrically defined by the pair of triangular facets adjacent to the crease and the dihedral angle between them. The crease element must describe not only the relative rotation between the two panels, but also the bending‐moment coupling between the crease and the neighboring panel deformation.

For this purpose, the crease contribution is decomposed into two parts: a rotational element and an additional bending element. The rotational element governs the relative rotation between the two panels through a virtual fold angle $\varphi_{v}$. As illustrated in Figure 1c, $\varphi_{v}$ is defined from the virtual tangential directions of the underlying smooth surface on the two sides of the crease. It represents the smooth‐surface folding angle, in contrast to the mesh dihedral angle φ, which is determined by the tangential directions of the adjacent triangular element. For a discrete crease segment of length $∥e_{i}∥$, the rotational energy is written as

(7) $$U_{\mathrm{rotation}}=\frac{1}{2}K_{r}{\left(\varphi_{v}-\varphi_{0}\right)}^{2}∥e_{i}∥,$$

where $K_{r}$ is the rotational stiffness and $\varphi_{0}$ is the rest fold angle. For a crease composed of multiple segments, the total rotational energy is obtained by summing Equation (7) over all crease segments.

In addition to the rotational stiffness, an additional bending element is introduced to account for the geometric discrepancy between the smooth‐surface fold representation and the mesh‐based fold representation. The virtual fold angle $\varphi_{v}$ defines a stress‐free bending configuration, whereas the mesh dihedral angle φ describes the actual discrete geometry near the crease. Their mismatch introduces an additional curvature contribution, which is added to the shell bending strain and thereby couples localized crease rotation with distributed panel bending.

This crease construction is embedded in the same DDG‐based geometric framework as the shell formulation, where element‐based metric/curvature measures and dihedral‐based crease measures are used consistently. Thus, the shell element, rotational crease element, and additional bending element share a unified geometric representation. Detailed definitions of the virtual tangential directions, the dihedral‐based curvature measure, and the additional bending strain are provided in the Section S1.3.

### 2.4 Environmental Interactions and Active Forces

In addition to the internal elastic response arising from panel deformation and crease mechanics, the robotic operation of compliant origami systems requires interactions with the external environment. In this work, the dynamic framework incorporates four representative external effects: gravitational loading, normal contact, tangential friction, and magnetic actuation. These contributions are assembled into the generalized external force vector as

(8) $$f_{\mathrm{ext}}=f_{g}+f_{\mathrm{contact}}^{N}+f_{\mathrm{contact}}^{T}+f_{\mathrm{mag}}.$$

Gravity is applied as a body force through the lumped mass of the shell vertices. Normal contact is modeled using a penalty‐based formulation to prevent interpenetration between the origami sheet and rigid obstacles. Tangential friction is described by a regularized Coulomb‐type law, which provides smooth behavior near zero sliding velocity and enables stable implicit time integration. Magnetic actuation is introduced through the interaction between magnetized shell faces and a prescribed external magnetic field [44, 45], with the corresponding nodal forces obtained from the derivative of the magnetic potential energy.

These environmental and active forces are applied to the physical shell vertices or panels, while the virtual crease variables are affected indirectly through their coupling with the shell deformation. Detailed formulations of gravity, contact regularization, friction, magnetic actuation, and their consistent tangent operators are provided in the Section S1.4.

## 3 Programmable Stability in Compliant Origami Units

In compliant origami systems, mechanical response is governed not only by kinematics but also by the competition between panel bending, crease rotation, and geometric constraints. This energetic interplay produces nonlinear stiffness variation, deformation mode selection, and bifurcation phenomena absent in purely rigid models. Within the proposed DDG framework, crease and panel stiffness are explicit and independently tunable parameters, making stability a programmable structural property rather than a fixed geometric outcome.

In this section, we investigate how the balance between crease rotational energy and panel bending energy governs the nonlinear response of representative origami units. We first analyze a minimal single‐fold (Z‐fold) configuration under tensile loading to isolate bending–crease coupling, and then examine shear‐induced nonlinear response and bifurcation behavior, demonstrating how stiffness‐controlled energy competition drives structural transitions.

### 3.1 Tensile Loading: Bending–Crease Coupling

**FIGURE 2:**
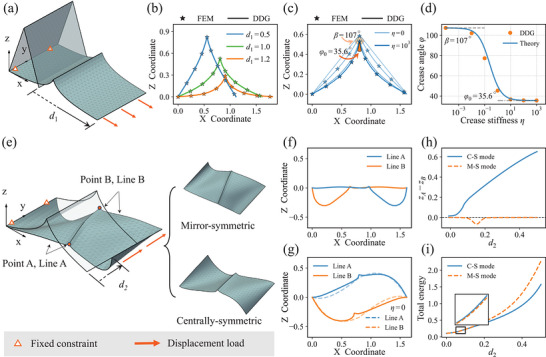
Mechanical response of the single‐fold structure. (a) Deformation configuration under tensile displacement loading. (b,c) Boundary profile evolution for varying applied displacement $d_{1}$ and different crease stiffness values. (d) Variation of the folding angle as a function of crease stiffness. (e) Two distinct deformation modes under shear displacement loading: mirror‐symmetric and centrally‐symmetric configurations. (f,g) Deformed boundary profiles corresponding to the two modes. (h,i) Evolution of nodal displacement and total energy in the two modes.

As shown in Figure 2a, a single‐fold structure consists of two deformable panels connected by a shared edge that defines a folding crease. In the discrete model, the crease is represented by a sequence of identical rotational elements associated with the mesh, allowing elastic deformation both within the panels and along the crease. The structure is subjected to tensile loading by prescribing displacements in the x‐direction at one end. Deformation profiles at loading lengths $d_{1}=0.5$, 1.0, and 1.2 are extracted and compared with FEM results under identical conditions. As shown in Figure 2b, the DDG‐based predictions exhibit excellent agreement with the FEM simulations.

By varying the ratio between hinge stiffness and panel bending stiffness, this example illustrates how the proposed DDG framework transitions continuously between rigid origami behavior and flexible deformation. Figure 2c shows the deformation profiles of the single‐fold structure for different crease stiffness values. To characterize the coupling between crease rotation and panel bending, we introduce a dimensionless stiffness ratio

(9) $$\eta =\frac{K_{r}W}{Eh^{3}},$$

where $K_{r}$ denotes the rotational stiffness density of the discrete crease, W is a characteristic length approximately equal to the panel size perpendicular to the hinge, and E and h are the Young's modulus and thickness of the panels. For η≪1, deformation localizes at the crease and the response approaches the rigid origami limit. For η≫1, crease rotation is suppressed and deformation occurs primarily through distributed panel bending. In both regimes, the DDG predictions remain in excellent agreement with FEM.

This transition is further quantified by examining the fold angle as a function of η (Figure 2d), which exhibits two plateau regimes. For small η, the fold angle is governed by geometric compatibility and corresponds to a rigid kinematic folding mode, yielding $\beta =107^{\circ }$. For large η, the fold angle approaches the initial value $\varphi_{0}=35.6^{\circ }$, indicating that deformation is mainly accommodated by panel bending. Between these limits, both mechanisms contribute, resulting in a smooth transition between the two plateaus.

To capture this energy competition, a simplified energy‐based Ritz model is developed (see Section S1 for details). The resulting closed‐form expression for the fold angle is

(10) $$\varphi =\beta +\frac{\eta (\varphi_{0}-\beta )}{1/2+\eta },$$

which explicitly describes the balance between crease stiffness and panel bending rigidity. In the limit η→0, φ=β, corresponding to pure kinematic folding, whereas η→∞ yields $\varphi =\varphi_{0}$, where the crease remains near its initial configuration. As shown in Figure 2d, the theoretical prediction agrees well with the numerical results.

This example demonstrates that the proposed DDG framework recovers rigid origami behavior as a limiting case while naturally capturing flexible deformation within a unified formulation.

### 3.2 Shear Loading: Nonlinear Response and Bifurcation

Further case studies illustrate the capability of the proposed framework in capturing nonlinear elastic behavior. As shown in Figure 2e, after the initial tensile loading, additional displacements $d_{2}$ are applied in the y‐direction. Under the combined effects of bending and shear, the structure exhibits two stable post‐buckling modes: a mirror‐symmetric mode and a centrally symmetric mode.

Deformation profiles along the two edges highlighted in blue and orange are extracted to characterize these modes. As shown in Figure 2f,g, the mirror‐symmetric mode produces edge profiles that are reflections of each other about the central axis in the x‐direction, whereas the centrally symmetric mode exhibits point symmetry about the structural center. Figure 2h plots the difference in the z‐coordinates of two crease points, A and B, during loading. In the mirror‐symmetric mode the two points remain at nearly the same height, while in the centrally symmetric mode their height difference increases with loading. The bifurcation between the two modes occurs immediately after the $d_{2}$ loading begins.

The activation of these modes depends on crease rotational stiffness and initial perturbations. Without crease stiffness, the centrally symmetric mode is the unique stable deformation path  [46]. Introducing finite crease stiffness promotes the mirror‐symmetric mode, as the crease constrains bending orthogonal to its direction and suppresses the development of the centrally symmetric pattern. Nevertheless, the mirror‐symmetric mode is not globally stable. Figure 2i compares the total energy evolution of the two modes. Although the mirror‐symmetric mode has slightly lower energy during the early stage of loading, the centrally symmetric mode maintains lower energy for most of the deformation and therefore represents the energetically preferred configuration. When a small perturbation breaks the symmetry at the crease, the structure ultimately transitions to the centrally symmetric mode, confirming it as the globally stable post‐buckling state.

These results demonstrate that stability characteristics can be tuned through crease stiffness modulation, providing the mechanical basis for the programmable robotic behaviors explored in the following sections.

## 4 Bridging Rigid and Compliant Origami Regimes

The design of compliant origami robots often spans a broad range of structural flexibility, from nearly rigid panels to highly compliant membranes. Accurate prediction of their mechanical response therefore requires a modeling framework capable of capturing both rigid folding and large‐deformation compliant behavior within a unified formulation. Miura origami provides an ideal testbed for this purpose  [47].

Despite sharing a single geometric pattern, Miura structures have been realized as rigid kinematic metamaterials  [31], moderately flexible systems for programmable surface shaping  [48], and highly compliant membranes for deployable aerospace structures  [18]. These realizations exhibit distinct deformation mechanisms due to differences in structural flexibility. In this section, we examine Miura origami across this stiffness spectrum through three representative cases: (i) rigid kinematic folding, (ii) out‐of‐plane bending in moderately flexible configurations, and (iii) large‐deformation deployment of highly compliant membranes. Together, these studies demonstrate that the DDG framework consistently captures both rigid and compliant behaviors within a single formulation.

### 4.1 Kinematic Folding in the Rigid Limit

**FIGURE 3:**
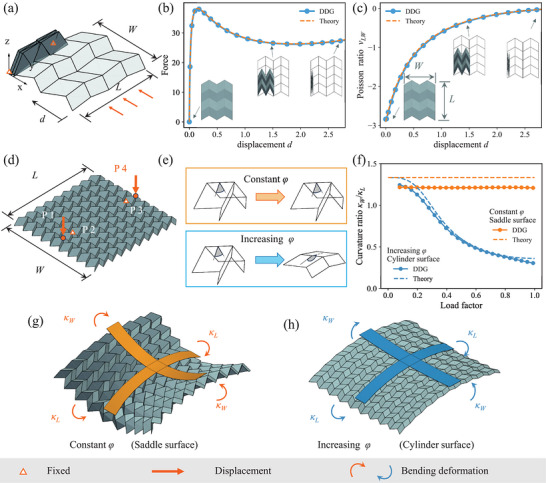
Mechanical behavior of rigid and flexible Miura origami structures. (a) Compressive deformation of the rigid Miura configuration, and (b) corresponding load–displacement response. (c) Evolution of the effective in‐plane Poisson's ratio. (d) Four‐point bending configuration applied to the flexible Miura structure. The theoretical model is derived from Ref. [31, 49]. (e) Evolution of the folding angle. (f) Comparison between the simulated curvature ratio and the analytical prediction. (g,h) Two deformed configurations under bending, exhibiting saddle‐shaped and cylindrical surface morphologies.

As illustrated in Figure 3a, we first consider the rigid‐origami limit using a structure composed of four Miura unit cells with a small dimensionless stiffness ratio η, indicating that crease stiffness is much smaller than panel bending stiffness.

A quasi‐static compression is applied along the x‐direction by fixing the left boundary (x=0) and prescribing a displacement to the nodes at the right boundary toward the negative x‐direction. Starting from an almost flat configuration, the reaction force at the right boundary is recorded during compression. The resulting load–displacement curve is shown in Figure 3b. After an initial high‐stiffness regime, the structure rapidly enters a negative‐stiffness region before the stiffness gradually increases as folding progresses. The DDG predictions agree closely with analytical kinematic models  [31, 49], validating the framework in the rigid‐origami limit. The simulations also reproduce the well‐known negative Poisson's ratio of Miura origami, where the structure contracts simultaneously in both the x‐ and y‐directions, as shown in Figure 3c.

### 4.2 Elastic Curvature Programming in Moderately Flexible Miura

Kinematic analyzes of rigid Miura origami indicate that its deformation is restricted to in‐plane stretching and compression modes. However, real sheet materials possess finite bending compliance, which introduces additional degrees of freedom and enables out‐of‐plane deformation.

Previous studies showed that when a Miura sheet is subjected to a uniaxial bending moment, it bends simultaneously in two orthogonal directions [31, 49]. This curvature coupling is known as the *bending Poisson effect*. To investigate this behavior, a four‐point bending configuration is applied to the Miura structure shown in Figure 3d, while the panel bending stiffness is reduced to allow out‐of‐plane deformation. Displacement constraints in the z‐direction are imposed at points $P_{2}$ and $P_{3}$, while prescribed downward displacements are applied at points $P_{1}$ and $P_{4}$.

A representative result is shown in Figure 3g. The structure bends along the loading direction L and simultaneously develops opposite curvature along the transverse direction W, forming a saddle‐shaped surface. The bending Poisson ratio is defined as $\nu_{b}=-\kappa_{W}/\kappa_{L}$, which geometric analysis predicts to equal the negative in‐plane Poisson ratio of the Miura pattern [49],

(11) $$-\kappa_{W}/\kappa_{L}=-\nu_{WL}.$$

Because the in‐plane Poisson ratio of Miura origami is negative, $\kappa_{W}$ and $\kappa_{L}$ have opposite signs, leading naturally to saddle‐shaped bending.

Beyond this classical response, the simulations also reveal an alternative deformation mode in which the structure evolves toward an approximately cylindrical surface (Figure 3h). The key difference between the saddle‐shaped configuration (Figure 3g) and the cylindrical one lies in the evolution of the fold angle φ. Accordingly, two regimes can be identified: a *constant‐* φ regime and an *increasing‐* φ regime, as illustrated in Figure 3e.

When crease stiffness is sufficiently large, panel bending dominates and the fold angles remain nearly constant, producing the saddle‐shaped mode. For smaller crease stiffness, deformation is governed primarily by fold rotation, leading to increasing φ and a cylindrical bending mode. Figure 3f shows the evolution of the bending Poisson ratio in both regimes. In the constant‐φ case, the ratio remains nearly constant and approaches the theoretical prediction $-\kappa_{W}/\kappa_{L}\approx 1.3$. In contrast, in the increasing‐φ regime the structure gradually unfolds and the bending Poisson ratio decreases toward 1/3, corresponding to nearly cylindrical deformation.

It is important to note that directly applying the closed‐form [49] prediction derived under the constant‐φ assumption to the increasing‐φ regime leads to discrepancies. This deviation does not indicate a breakdown of the geometric relation, but rather reflects the fact that the fold angle evolves during deformation and therefore cannot be treated as constant, as shown in Figure 3f. When the instantaneous fold angle φ obtained from simulations is substituted into the analytical expression for the bending Poisson ratio, the theoretical prediction recovers excellent agreement with the numerical results. This confirms that the geometric relation in Equation (11) remains valid throughout the deformation process, provided that the effective Poisson ratio $\nu_{WL}$ is evaluated using the evolving fold angle φ.

Under large bending deformations, the Miura array develops spatially nonuniform curvature fields, and the fold angles vary both temporally and spatially across the structure. These nonlinear effects limit the applicability of closed‐form solutions based on fixed geometric parameters  [15]. The numerical framework proposed in this work therefore provides a consistent means of tracking the evolution of fold angles and curvature, enabling accurate prediction of the global mechanical behavior of Miura origami.

### 4.3 Highly Flexible Deployable Membrane Behavior

Miura origami structures are widely used in aerospace applications due to their excellent deployability, particularly for ultra‐thin membrane systems requiring in‐orbit deployment. Such systems combine high membrane flexibility with crease constraints, leading to strongly nonlinear responses during deployment  [17]. The crease–membrane coupled framework developed in this work is therefore well suited for simulating the deployment behavior of highly flexible Miura membranes.

**FIGURE 4:**
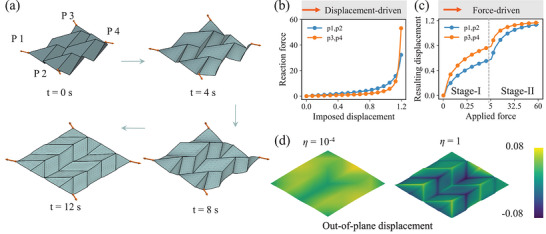
Deployment simulation of Miura origami membranes. (a) Evolution of the deployed configuration at representative time instants. The deployable membrane configuration is derived from Ref. [17]. (b,c) Load–response curves under displacement‐controlled and force‐controlled actuation, respectively. (d) Influence of crease stiffness on the surface flatness of the deployed membrane.

To represent typical actuation strategies, both displacement‐driven and force‐driven loading schemes are considered, corresponding to cable‐driven and thruster‐driven deployment. In the numerical model, loads are applied at the four corner points P1–P4 (Figure 4a). For displacement‐driven deployment, constant‐velocity displacements of 0.1/s are prescribed along the directions connecting the initial and fully deployed positions. The resulting load–displacement response (Figure 4b) reveals two distinct mechanical stages. In the first stage (0–8s), deployment is mainly accommodated by panel bending, resulting in relatively low stiffness and gradually increasing reaction forces. In the second stage, deformation is dominated by fold‐angle changes as the structure approaches a planar configuration. As the geometry flattens, the effective moment arm of the external loads decreases, requiring significantly larger forces to drive further crease rotation and producing a pronounced stiffening response.

For force‐controlled deployment (Figure 4c), a staged loading strategy is adopted based on the two‐stage behavior observed above. The forces at P1–P4 are equal in magnitude and increased from 0 to 5 in the first stage and from 5 to 60 in the second stage. This loading protocol mitigates excessive variations in the deployment rate and enables stable deployment throughout the process. The simulations demonstrate that the proposed framework robustly captures the strongly nonlinear response, providing a reliable basis for the design and optimization of deployment actuation strategies.

Because the creases possess finite stiffness, the final deployed configuration is not perfectly flat but results from the coupled effects of membrane bending and crease constraints. Figure 4d compares deployed shapes for two representative stiffness ratios, $\eta =1\times 10^{-4}$ and η=1. For larger crease stiffness, pronounced surface nonuniformity remains even after full deployment. This behavior cannot be captured by conventional models that neglect crease stiffness, highlighting the importance of crease–membrane coupled modeling for highly flexible deployable structures  [18].

### 4.4 Model Applicability and Computational Efficiency

The three examples in this section represent deformation regimes that are traditionally treated by different modeling strategies: rigid‐folding kinematics for ideal crease rotation, reduced bar‐hinge models for moderately flexible origami with limited panel deformation, and continuum shell finite element simulations for highly flexible structures with distributed stretching, bending, twisting, and nonuniform curvature. The proposed DDG framework unifies these regimes within the same origami geometry, enabling a continuous exploration from rigid‐like folding to highly compliant deformation.

Compared with shell FEA, the DDG model reduces the number of unknowns in the nonlinear system and thereby improves the per‐iteration computational efficiency, as shown in Tables S1 and S2. In addition, mesh‐refinement tests show that the DDG solution converges steadily with a trend comparable to low‐order shell FEM (Figure S1), indicating that the model can recover increasingly resolved continuum‐shell‐like deformation as the mesh is refined. This allows coarse meshes for rapid exploration and refined meshes for more accurate simulations of complex compliant deformation mode.

The proposed method is not intended as a high‐fidelity replacement for shell FEA in all scenarios. Rather, it provides a lightweight continuum‐shell‐like model that can be coarsened for efficient bar‐hinge simulations or refined toward shell‐like accuracy. This capability is useful for design‐oriented studies of compliant origami robots, where deformation modes, stability transitions, snap‐through, and actuation responses need to be examined within the same framework.

## 5 From Structural Mechanics to Robotic Functionality

Building upon the structural analyzes presented above, we further extend the framework to origami‐inspired compliant robotic systems, where mechanical response directly translates into functional motion. Two representative examples are considered: a jumping robot based on the bistable behavior of the Waterbomb unit, and a crawling robot driven by the directional actuation of a Kresling mechanism. In next two sections, we examine how these structural principles inform robotic design, including actuation strategies and motion generation, and demonstrate that the proposed DDG framework enables provides a consistent dynamic modeling framework for simulating complex robotic motion under nonlinear and multistable structural responses.

### 5.1 Jumping Robot: Bistable Actuation in Waterbomb Origami

The Waterbomb origami is a classical folding pattern characterized by an alternating arrangement of mountain and valley folds around a central vertex [32, 50]. Owing to its geometric nonlinearity, this configuration is known to exhibit bistable behavior [51, 52]. In this section, two numerical case studies are presented to investigate the bistability of Waterbomb structures, considering (i) a flexible waterbomb and (ii) a rigid waterbomb. For both cases, the proposed framework successfully captures the existence of two stable equilibrium states, as well as the snap‐through behavior associated with dynamic transitions between them.

#### 5.1.1 Bistable of Flexible Waterbomb

**FIGURE 5:**
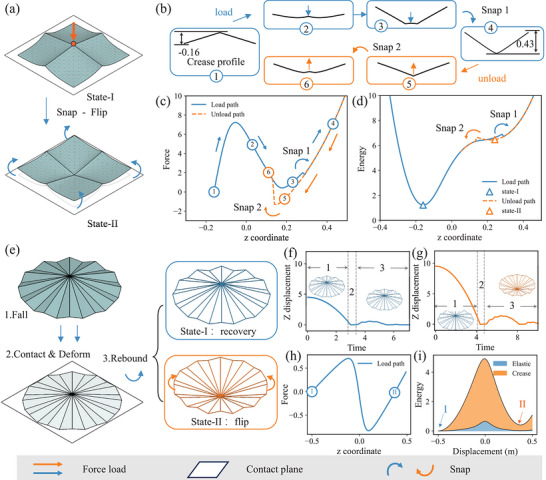
Bistable mechanical behavior of the Waterbomb origami. (a) Two stable states of the flexible Waterbomb configuration and the associated flip. (b) Deformation profiles at representative instants during the state‐switching process. (c,d) Load–displacement response and corresponding energy evolution. (e) Two stable states of the rigid Waterbomb configuration [53] and its snap‐through behavior. (f,g) Time–displacement histories under free fall from heights z=5 and z=10, leading to recovery and flip, respectively. (h,i) Quasi‐static load–displacement response and energy distribution.

The flexible case is constructed from a simple origami configuration. Starting from a square sheet, the paper is folded along two symmetry axes parallel to the edges, forming four mountain creases with intrinsic fold angles induced by plastic deformation (Figure 5a). For a purely rigid origami model, a unit consisting only of four mountain folds cannot deform compatibly, as this would require an isometric mapping from a plane to a surface with nonzero Gaussian curvature, violating Gauss' Theorema Egregium  [47]. When material flexibility is introduced, this geometric incompatibility can be accommodated through panel bending. As shown in Figure 5a (state–I), spontaneous bending develops in the central region enclosed by the creases, effectively acting as a virtual valley fold and producing a flexible Waterbomb configuration. When placed on a flat surface, pressing the center induces an up–down inversion of the boundary, and the inverted configuration (state–II) remains stable after unloading, demonstrating bistability.

To capture this behavior, dynamic simulations are performed using the proposed framework. The model consists of four flexible panels connected by four creases and interacting with a rigid horizontal plane. Gravity and normal contact with the supporting surface are included. Intrinsic fold angles generate the initial bent configuration (state–I), where the four corners serve as stable supports. A downward force applied at the center then drives the structure to invert and transition to the second stable state (state–II).

To further analyze the equilibrium behavior, quasi‐static simulations are conducted by prescribing vertical displacement at the center. The resulting crease‐edge profiles and load–displacement response at representative configurations are shown in Figure 5b,c. Along the loading path (blue curve), the structure initially exhibits a short regime of positive stiffness, followed by a negative stiffness region and subsequently a return to positive stiffness as the deformation proceeds from configuration 1 to 4. During this process, the relative height between the center and the boundary undergoes a reversal. Notably, the crease‐edge profile at configuration 3 is not smooth, suggesting that this state corresponds to an unstable equilibrium. As a result, the structure undergoes a small‐amplitude snap‐through transition from configuration 3 to 4, recovering stability. Configuration 4 coincides with the second stable equilibrium (state–II). This snap‐through event is manifested as a discontinuous jump in the load–displacement curve and is denoted as snap 1. After reaching the second stable state, the structure is unloaded, as indicated by the orange curve Figure 5c. Owing to the asymmetry between the two stable configurations, the unloading path does not retrace the loading path. Instead, the structure deforms stably over a finite range and, while remaining in the state–II configuration, passes beyond z=0.2. After configuration 5, a second snap‐through event (snap 2) occurs, driving the system back to the primary equilibrium branch.

The mechanism of these snap events is clarified by the energy curves in Figure 5d. The two stable configurations correspond to local minima on different energy branches: the loading branch defines state–I, whereas the unloading branch defines state–II. Because these branches do not coincide, the system must leave the loading path and relax to the unloading branch once it passes the vicinity of state–II, giving rise to snap 1. A similar mechanism produces snap 2 during unloading.

#### 5.1.2 Jumping Behavior of the Rigid Waterbomb

The bistability of the Waterbomb structure enables energy storage during transitions between its two stable states, which can be exploited to realize a jump origami robot [53]. To illustrate the capability of the proposed framework in capturing the dynamics of such a system, we perform numerical simulations based on the mechanism described in the literature.

The robot model consists of a rigid Waterbomb origami with 12 mountain and valley creases, exhibiting two stable configurations: the initial configuration (state–I) and its inverted configuration (state–II), as shown in Figure 5e. When released from a certain height, the robot undergoes three sequential stages: free fall, deformation, and energy storage upon contact with the ground, and subsequent rebound. Depending on the gravitational potential energy imparted during the fall, the robot may either recover its initial configuration (recovery state–I) or transition to the inverted configuration (state–II) after the rebound. Figure 5f,g depicts the trajectories of the Waterbomb robot released from heights of z=5 and z=10, respectively. After completing the three stages, the robot released from z=5 does not acquire sufficient energy to overcome the energy barrier and returns to state–I, whereas the robot released from z=10 successfully transitions to state–II.

The load–response behavior of the rigid Waterbomb robot is shown in Figure 5h. The curve exhibits a sequence of positive stiffness, negative stiffness, and positive stiffness during the transition between stable states, similar to the corresponding response of the flexible robot (Figure 5c). Compared with the flexible system, the rigid robot displays a smoother transition between stable states, avoiding the small‐amplitude snap events observed in the flexible case. The corresponding energy distribution during state transitions is illustrated in Figure 5i, indicating that the deformation energy in the rigid origami is primarily concentrated in the creases. By applying external forces to drive the robot over the energy barrier, the input energy is temporarily stored in the structural deformation and subsequently converted into kinetic energy, thereby enabling the jump mechanism [53].

### 5.2 Crawling Robot: Directional Actuation in Kresling Origami

The Kresling origami pattern is a cylindrical shell structure with intrinsic multistability. Its geometry is defined by the intersections of two families of longitudinal helical creases and a set of transverse circular creases. Due to its axial stackability, multiple units can be assembled to enable directional actuation. Leveraging this property, the elegant compact magnetically actuated soft origami robot developed in Ref. [8] achieves remarkably precise controllable in‐plane contraction and directional locomotion through externally applied magnetic fields. In this section, the proposed unified DDG‐based framework is used to simulate the complex nonlinear dynamics of this Kresling‐based robotic system.

#### 5.2.1 Multistability of the Rigid Kresling System

**FIGURE 6:**
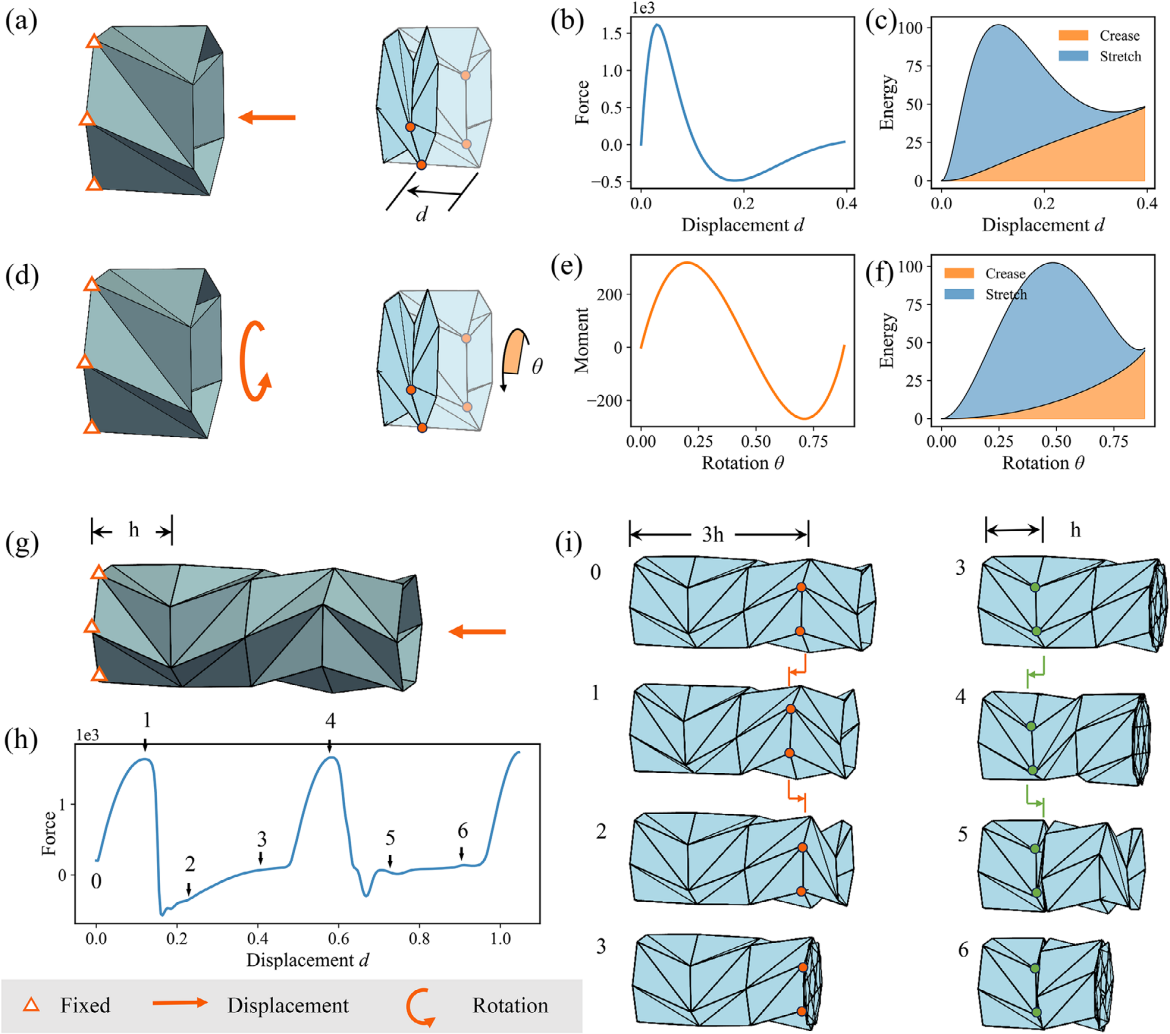
Multistable behavior of the rigid Kresling origami. (a–c) Load–displacement response and corresponding energy landscape of a single rigid Kresling unit under axial displacement loading. (d–f) Load–displacement response and energy distribution under circumferential rotational displacement loading. (g) Dynamic switching simulation of a four‐unit rigid Kresling assembly exhibiting multistable transitions. (h) Time histories associated with the dynamic switching process. (i) Deformed configurations at representative time instants during multistable transition.

For an individual Kresling unit, fundamental folding and deployment can be achieved either by axial compression or by prescribing a relative rotation between the upper and lower annular faces (Figure 6a,d). Figure 6b,c shows the force–displacement response under axial loading and the corresponding energy evolution. The structure exhibits a characteristic positive–negative–positive stiffness transition, indicating bistability: reaction forces vanish at both the fully deployed (d=0) and fully folded (d≈0.4) configurations, corresponding to local minima in the potential energy. Unlike the Waterbomb structure, where bistability arises from panel bending–crease coupling, the Kresling unit's multistability is primarily driven by panel stretching induced by crease‐length variations. During the state transition, stretching energy rises to a peak at the energy barrier and then decreases, dominating the overall energy evolution. Figure 6e,f shows that circumferential torsional loading also produces bistable behavior with a similar mechanism.

Stacking four Kresling units axially forms a multi‐unit structure with complex multistability. Dynamic simulations (Figure 6g) reveal pronounced nonlinear state‐switching under right‐end compression with the left end fixed. The sum of reaction forces vs. displacement is plotted in Figure 6h, and six representative configurations in Figure 6i illustrate the transition sequence.

The first complete stable‐state transition (configurations 0–3) occurs in three stages: **In stage 0–1,** the imposed displacement simultaneously compresses all four units, leading to axial shortening in each. The combined length of the bottom three units gradually decreases to below 3h. **In stage 1–2,** When the reaction force reaches the critical peak value ($F\approx 1.5\times 10^{3}$), the bottom three units undergo snap‐back instability and rapidly return to their original combined length of 3h, while the outermost unit loses stiffness and is quickly compressed. **In stage 2–3,** The outermost unit continues to compress until it becomes fully folded.

Stages 3–6 repeat this sequence, completing a second stable‐state transition. After two cascaded transitions, the four‐unit stack is compressed to a total length of two unit heights. This cascade‐like behavior, triggered by local unit instability, highlights the strongly nonlinear and path‐dependent response of the stacked Kresling system.

#### 5.2.2 Dynamics of a Compliant Crawling Robot

**FIGURE 7:**
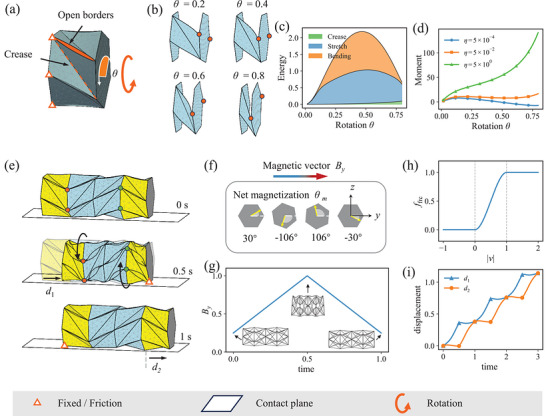
Dynamic locomotion of a compliant crawling robot actuated by Kresling‐based units. (a–d) Mechanical characteristics of a compliant Kresling actuator, including its load–displacement response and multistable behavior. (e) One complete locomotion cycle of the crawling robot [8]. (f) Schematic of the external magnetic field and orientation of the net magnetization vector in the actuator. (g) Robot configurations under varying magnetic field strengths. (h) Definition of the friction coefficient governing foot–ground interaction. (i) Time histories of the foot displacement during robot locomotion.

The Kresling origami structure enables directional actuation via axial stacking, but its intrinsic bistability can cause abrupt snap‐through transitions, challenging controllability. To improve performance, compliant Kresling designs have been proposed  [22]. Following this strategy, all mountain creases are removed to create open borders that can deform freely (Figure 7a), while the remaining creases are stiffened to enhance rotational constraint.

Simulations of the compliant Kresling mechanism reveal that, under rotational loading of the right end face, geometric incompatibility is accommodated via bending concentrated near the open borders, replacing the stretching‐dominated response of the rigid configuration(Figure 7b). This mechanism substantially lowers the energy barrier between stable states, as shown in Figure 7c, primarily by reducing panel stretching energy. The torque–rotation response (Figure 7d) shows that at low crease stiffness, the structure remains bistable with a non‐monotonic curve. Increasing the stiffness gradually eliminates snap‐through, producing a monotonic, stable response conducive to precise actuation This demonstrates the advantages of the programmable stability of compliant origami.

Building on this design, a compliant origami robot  [8] is modeled as four Kresling units stacked in alternating symmetric and antisymmetric configurations (Figure 7e). Magnetic plates attached to transverse panels generate torsional deformation under an external field, which is converted into axial extension and contraction via Kresling kinematics. Directional crawling is achieved through anisotropic foot friction.

The robot simulation accounts for elastic and kinetic energies, magnetic forces, gravity, contact, and friction. As illustrated in Figure 7f, each magnetized panel has a vector inclined at $\theta_{m}$ to the y‐axis, with a uniform field applied along y. Varying the field strength controls the global configuration: weak fields restore the extended state, strong fields induce folding (Figure 7g). As an additional validation, we compare the DDG predictions with the experimental results reported in Ref.  [8]. The comparison, summarized in Section S5, includes the force–displacement and stored‐energy–displacement responses of the soft origami actuation unit, together with the magnetically driven response of the assembled robot.

Forward locomotion is achieved through directional (anisotropic) friction at the robot feet. Specifically, a preferred unit direction vector t is assigned at each foot contact, defined as the direction perpendicular to the foot‐ground contact segment and oriented along the forward locomotion direction. The directional friction force is evaluated using the regularized Coulomb model, and only its projected component along t, i.e., $f_{\mathrm{contact}}^{t}=\left(f_{\mathrm{contact}}^{T}\cdot t\right)t$, is retained to generate directional frictional response. A unidirectional, $C^{1}$‐continuous friction regularization function is applied at foot contacts (Figure 7h):

(12) $$f_{\text{frc}}\left(|v|\right)=\left\{\begin{array}{cc} 0, & |v|<0,\\ {3|v|}^{2}-2{\left|v\right|}^{3}, & 0\leq |v|<1,\\ 1, & |v|\geq 1, \end{array}\right.$$

where $\left|v\right|=\left(\dot{v}\cdot t\right)/\varepsilon_{v}$ is the normalized tangential velocity component along the preferred friction direction. This formulation ensures smooth transitions across no‐, static‐, and kinetic–friction regimes while introducing directional locomotion selectivity.

Figure 7i presents the time histories of the horizontal displacements of two representative foot nodes. Each crawling cycle consists of two stages. During the first stage (0–0.5s), the magnetic field strength increases, causing the robot to fold. In this phase, leftward motion of the feet is restricted by friction, while rightward motion remains unimpeded. As a result, the right foot displacement $d_{2}$ remains nearly zero, whereas the left foot displacement $d_{1}$ increases toward the right. During the second stage (0.5–1s), the magnetic field decreases and the robot re‐extends. The frictional constraint reverses: $d_{1}$ becomes fixed, and $d_{2}$ advances to the right. Through this alternating deformation and frictional rectification mechanism, the robot achieves net forward crawling over each cycle.

## 6 Conclusion

In this work, we established a unified DDG–based framework for the simulation of robotic origami system spanning the full spectrum from rigid kinematic folding to compliant shell deformation. Motivated by the need to both understand and engineer compliant origami robots, the proposed formulation bridges traditionally separated modeling paradigms—rigid‐folding kinematics, bar‐and‐hinge abstractions, and continuum shell mechanics—within a single geometrically consistent structure.

By introducing shell and crease elements under a shared mid‐edge discretization, and by expressing their mechanics through dual face– and dihedral‐angle–based representations, the framework provides a coherent description of bending, folding, and rotation in a unified variational setting. This consistency enables rigid motion, elastic deformation, and nonlinear stability phenomena to emerge as different regimes of the same underlying formulation, rather than as fundamentally distinct models. To capture the strongly nonlinear and dynamic behaviors inherent to origami systems, an implicit time integration scheme and environmental interactions—including gravity, contact, friction, and magnetic actuation—were incorporated into the framework. The resulting model enables the systematic investigation of multi‐stability, snap‐through transitions, frictional contact, and multiphysics coupling within a robust computational environment.

Through a progressive set of representative examples—including the single‐fold (Z‐fold), the Miura pattern, the Waterbomb pattern, and the Kresling structure—the framework was validated across increasing levels of geometric complexity and mechanical nonlinearity. These examples span a spectrum from fundamental folding units to functionally actuated origami robots, covering behaviors ranging from simple geometric reconfiguration to snap‐through propulsion and directional locomotion. For each case, rigid and compliant formulations were examined within the same DDG structure, revealing how mechanical parameter tuning continuously reshapes the system's response landscape. These results demonstrate not only the accuracy and robustness of the approach, but also its capacity to expose the underlying mechanics that govern programmable folding behavior.

Overall, this work provides both a conceptual and computational foundation for compliant dynamic origami systems. By unifying geometry, elasticity, and dynamics in a single formulation, the framework advances the theoretical understanding of compliant origami mechanics while simultaneously offering a practical design and analysis tool for next‐generation programmable origami robots. The present DDG framework is well suited to origami systems with coupled in‐plane panel deformation and out‐of‐plane folding, and therefore may be extended to more complex origami architectures that are particularly attractive for robotic design, such as Yoshimura‐pattern origami robots with morphologically rich and functionally versatile deformation modes [54, 55]. This potential extension also identifies an aspect that requires further treatment in the current formulation: facet–facet self‐contact is not explicitly enforced, and folding closure is regularized primarily through the hinge‐angle penalty near the fully folded state. Therefore, highly compliant structures with extensive contact during folding would require additional contact detection and contact‐force treatment to ensure robust simulations. Future directions include inverse design strategies, optimization‐based pattern synthesis, and integration with model‐based control methodologies to further enable mechanically intelligent and adaptive origami robotic systems.

## Conflicts of Interest

The authors declare no conflicts of interest.

## Supporting information


**Supporting File 1**: advs76715‐sup‐0001‐SuppMat.pdf.

**Supporting File 2**: advs76715‐sup‐0002‐VideoS1.mp4.

## Data Availability

The data that support the findings of this study are available from the corresponding author upon reasonable request.
