# Supplementary material for: From Folding Mechanics to Robotic Function: A Unified Modeling Framework for Compliant Origami
Source: Adv Sci (Weinh). 2026 Jul 24:e76715. Online ahead of print. doi: 10.1002/advs.76715 (PMC13398139; doi:10.1002/advs.76715)
Supplement: Supplementary file 1 — Supporting File 1: advs76715‐sup‐0001‐SuppMat.pdf. [file ADVS-9999-e76715-s001.pdf]

Supplementary Information for *From Folding Mechanics to Robotic Function: A Unified Modeling Framework for Compliant Origami*

Bohan Zhang, Bo Wang, Huajiang Ouyang, Zhigang Wu, Haohao Bi, Jiawei Xu, Mingchao Liu*, Weicheng Huang**

Contents

S1 DDG-Based Formulation for Compliant Origami Robots 2
S1.1 Unified Geometric and Variational Formulation . . . 2
S1.2 Discrete Elastic Energy of Compliant Panels . . . 2
S1.3 Geometry-Consistent Modeling of Crease . . . 4
S1.4 Dynamic Formulation for Active Origami Systems . . . 5

S2 Ritz Approximation for the Folding Angle 8

S3 Finite element method (FEM) 10

S4 Model Applicability and Computational Efficiency 11

S5 Experimental Comparison of the Compliant crawling Robot Simulation 13

S6 Video 15

S1 DDG-Based Formulation for Compliant Origami Robots

S1.1 Unified Geometric and Variational Formulation

For realistic modeling of compliant origami robots, non-conservative external effects — including gravity, contact, friction, and field-driven actuation (e.g., magnetic forces) — must also be incorporated. The dynamic response of the origami structure is governed by

$$\mathbb{M}\,\ddot{\mathbf{q}}(t) + \mathbb{C}\,\dot{\mathbf{q}}(t) + \mathbb{K}\,\mathbf{q}(t) = \mathbf{f}_{\text{ext}}(t), \tag{S1}$$

where $\mathbb{M}$ is the mass matrix derived from the kinetic energy, $\mathbb{C}$ is the damping matrix accounting for energy dissipation, and $\mathbf{f}_{\text{ext}}(t)$ represents the externally applied forces. In Equation (S1), the generalized coordinate vector $\mathbf{q}(t)$ collects all discrete degrees of freedom (DOFs) of the origami system. As illustrated in Figure 1, a flexible origami structure consists of two fundamental modules: the shell module and the crease module. A minimal origami unit contains at least one crease set and two adjacent shell panels. The detailed discretization procedures of these two modules will be presented in the following sections. Here, we directly outline the structure of the generalized coordinates. The DOFs of the origami system consist of two distinct sets of variables. The first set corresponds to the translational degrees of freedom of the shell mesh, namely the spatial coordinates of all vertices,

$$\mathbf{v}_{(a)}(t) = \begin{bmatrix} x_{(a)}(t) & y_{(a)}(t) & z_{(a)}(t) \end{bmatrix}, \text{ with } a = 1, \ldots, N_1,$$

where N_1 denotes the total number of vertices. The second set comprises the virtual folding angles associated with each crease element,

$$\varphi_{v,(b)}(t), \text{ with } b = 1, \ldots, N_2,$$

where N_2 is the total number of creases. The generalized coordinate vector is therefore written compactly as

$$\mathbf{q}(t) = \left[\mathbf{v}_{(1)}(t), \ldots, \mathbf{v}_{(N_1)}(t), \varphi_{v,(1)}(t), \ldots, \varphi_{v,(N_2)}(t)\right]^{\mathsf{T}}, \tag{S2}$$

which yields a total of $3N_1 + N_2$ degrees of freedom. The configuration-dependent stiffness matrix $\mathbb{K}(\mathbf{q})$ is obtained from the second derivative of the elastic potential energy $\Pi(\mathbf{q})$ with respect to the generalized coordinates, namely

$$\mathbb{K}(\mathbf{q}) = \frac{\partial^2 \Pi(\mathbf{q})}{\partial \mathbf{q}^2}.$$

Owing to the geometric and material nonlinearities inherent in origami structures, $\mathbb{K}$ depends explicitly on the instantaneous configuration $\mathbf{q}(t)$ and is therefore continuously updated during deformation. Equation (S1) thus provides a unified formulation that accounts simultaneously for inertial, elastic, damping, and external effects within a unified nonlinear dynamic framework. Time integration is performed using an implicit Euler scheme, and the resulting nonlinear system is solved by Newton iterations. Although this scheme is robust, it introduces numerical dissipation, so smaller time steps are needed for highly dynamic snap-through events where inertial effects are significant. The source code of the simulation framework is publicly available in an open-access repository [1]. In the following, we detail the construction of the elastic energy terms and the treatment of environmental interactions.

S1.2 Discrete Elastic Energy of Compliant Panels

The deformable panels of the origami robot are modeled as thin shells, as illustrated in Figure 1(a). Within the simulation framework, each crease is treated as a free boundary, and the two panels adjoining a crease are regarded as independent shell bodies. Mechanical coupling between adjacent panels is introduced through dedicated crease elements. As shown in Figure 1(b), each shell panel is represented as a smooth surface discretized by a triangular mesh. To ensure a consistent interplay between geometry and mechanics, we adopt a discrete differential geometry (DDG) formulation [2, 3]. Under this framework, the surface is described by discrete analogues of the first and second fundamental forms, which

characterize in-plane metric distortion and out-of-plane curvature, respectively. These geometric descriptors provide the basis for defining membrane and bending strain measures in a manner that remains geometrically exact under large rotations and deformations.

To describe the deformation of each shell panel, consider a triangular element $\mathcal{T}_{ijk}$ with vertices i, j, and k, as shown in Figure 1(b). The shell panel is discretized into N_t such triangular elements. Both the discrete mesh and the associated smooth surface are embedded in $\mathbb{R}^3$. We use $\mathbf{e}_i$ denote the edge opposite to vertex i, and similarly for $\mathbf{e}_j$ and $\mathbf{e}_k$. If the discrete surface closely approximates the underlying smooth surface, the edge vectors provide finite-difference approximations of the tangent vectors of $\mathbf{r}_{ijk}$. The first fundamental form measures the metric of the surface, i.e., the inner product of tangent vectors. In the discrete setting, this metric is naturally defined through edge vectors. For an edge $\mathbf{e}_i$ opposite to vertex i, with vertex positions $\{\mathbf{v}_i, \mathbf{v}_j, \mathbf{v}_k\}$, the discrete first fundamental form is defined as [4]

$$\mathbb{A}(\mathbf{e}_i, \mathbf{e}_i) = (\mathbf{v}_k - \mathbf{v}_j) \cdot (\mathbf{v}_k - \mathbf{v}_j). \tag{S3}$$

Moreover, using the two edges emanating from vertex i, $\mathbf{e}_k$ and $-\mathbf{e}_j$, as a basis spanning the discrete tangent space of the triangular element $\mathcal{T}_{ijk}$, the discrete first fundamental form can be written in tensor form as

$$\mathbb{A}_{ijk} = \begin{bmatrix} (\mathbf{v}_j - \mathbf{v}_i) \cdot (\mathbf{v}_j - \mathbf{v}_i) & (\mathbf{v}_j - \mathbf{v}_i) \cdot (\mathbf{v}_k - \mathbf{v}_i) \\ (\mathbf{v}_k - \mathbf{v}_i) \cdot (\mathbf{v}_j - \mathbf{v}_i) & (\mathbf{v}_k - \mathbf{v}_i) \cdot (\mathbf{v}_k - \mathbf{v}_i) \end{bmatrix}. \tag{S4}$$

The second fundamental form characterizes the variation of the surface normal d$\mathbf{n}$ induced by tangential displacement and measures the bending of the surface. To construct its discrete counterpart, a discrete normal field $\mathbf{n}$ needs be defined. We adopt a *mid-edge discretization* in which the normal vector is associated with each edge midpoint [5, 6]. For an edge $\mathbf{e}_i$ of the triangular element $\mathcal{T}_{ijk}$, the associated mid-edge normal $\mathbf{n}_i$ is defined as follows: if $\mathbf{e}_i$ lies on the boundary of the structure, $\mathbf{n}_i$ coincides with the unit normal of the incident face,

$$\mathbf{n}_i = \frac{(\mathbf{v}_j - \mathbf{v}_i) \times (\mathbf{v}_k - \mathbf{v}_i)}{\|(\mathbf{v}_j - \mathbf{v}_i) \times (\mathbf{v}_k - \mathbf{v}_i)\|}, \tag{S5}$$

otherwise, for an interior edge, $\mathbf{n}_i$ is taken as the average of the unit normals of the two adjacent faces sharing $\mathbf{e}_i$. Within each triangular element, bending is evaluated through finite differences of the normal field along edge directions. Exploiting the fact that the segment connecting two edge midpoints is parallel to the opposite edge, the discrete second fundamental form along edge $\mathbf{e}_i$ is defined as

$$\mathbb{B}(\mathbf{e}_i, \mathbf{e}_i) := 2(\mathbf{n}_j - \mathbf{n}_k) \cdot (\mathbf{v}_k - \mathbf{v}_j). \tag{S6}$$

And the tensor representation of the discrete second fundamental form for $\mathcal{T}_{ijk}$ is written as

$$\mathbb{B}_{ijk} = 2 \begin{bmatrix} (\mathbf{n}_j - \mathbf{n}_i) \cdot (\mathbf{v}_j - \mathbf{v}_i) & (\mathbf{n}_j - \mathbf{n}_i) \cdot (\mathbf{v}_k - \mathbf{v}_i) \\ (\mathbf{n}_k - \mathbf{n}_i) \cdot (\mathbf{v}_j - \mathbf{v}_i) & (\mathbf{n}_k - \mathbf{n}_i) \cdot (\mathbf{v}_k - \mathbf{v}_i) \end{bmatrix}. \tag{S7}$$

In this manner, the discrete fundamental forms provide geometric measures of in-plane metric distortion and out-of-plane curvature variation for each triangular element. Given a reference configuration of the triangular mesh with vertex positions $\{\bar{\mathbf{v}}_i, \bar{\mathbf{v}}_j, \bar{\mathbf{v}}_k\}$, the corresponding reference first and second fundamental forms, $\bar{\mathbb{A}}_{ijk}$ and $\bar{\mathbb{B}}_{ijk}$, are constructed analogously. Within the Kirchhoff–Love shell assumption, the deformation of a triangular element $\mathcal{T}_{ijk}$ is characterized by two strain measures: the membrane (in-plane) strain and the bending (out-of-plane) strain. The discrete membrane strain is defined as the relative change of the metric,

$$\boldsymbol{\varepsilon}_{ijk} = \bar{\mathbb{A}}_{ijk}^{-1} \mathbb{A}_{ijk} - \mathbf{I}, \tag{S8}$$

which serves as a discrete analogue of the Green–Lagrange strain tensor [7]. The discrete bending strain is measured through the change of curvature,

$$\boldsymbol{\kappa}_{ijk} = \bar{\mathbb{A}}_{ijk}^{-1} \left(\mathbb{B}_{ijk} - \bar{\mathbb{B}}_{ijk} \right). \tag{S9}$$

The total elastic energy of the shell is then written as the sum of stretching and bending contributions,

$$U_{\text{shell}} = U_{\text{stretch}} + U_{\text{bend}}, \tag{S10}$$

with

$$U_{\text{stretch}} = \sum_{ijk}^{N_t} \frac{h}{8} \, \|\boldsymbol{\varepsilon}_{ijk}\|_{SV}^2 \, \sqrt{\det(\bar{\mathbb{A}}_{ijk})}, \tag{S11}$$

$$U_{\text{bend}} = \sum_{ijk}^{N_t} \frac{h^3}{24} \, \|\boldsymbol{\kappa}_{ijk}\|_{SV}^2 \, \sqrt{\det(\bar{\mathbb{A}}_{ijk})}. \tag{S12}$$

Here, h denotes the thickness of the shell and $\sqrt{\det(\bar{\mathbb{A}}_{ijk})}$ represents the reference area of the element. The operator $\|\cdot\|_{SV}^2$ indicates that the constitutive behavior follows the linear St. Venant–Kirchhoff model. For instance, the membrane strain $\boldsymbol{\varepsilon}_{ijk}$ is evaluated as

$$\|\boldsymbol{\varepsilon}_{ijk}\|_{SV}^2 = \frac{E\nu}{2(1-\nu^2)} \text{Tr}^2(\boldsymbol{\varepsilon}_{ijk}) + \frac{E}{2(1+\nu)} \text{Tr}(\boldsymbol{\varepsilon}_{ijk}^2), \tag{S13}$$

where E and ν denote the Young's modulus and Poisson's ratio, respectively.

S1.3 Geometry-Consistent Modeling of Crease

We next consider the discrete formulation of crease in a flexible origami system. Two independent shell panels are coupled along a prescribed crease line through a dedicated crease element. The crease element is designed to consistently bridge the kinematics of smooth surfaces and their discrete triangular representations. To this end, we decompose the crease contribution into two components: (i) a *rotational element*, which governs the relative rotation between adjacent panels, and (ii) an *additional bending element*, which accounts for the discrepancy between the discrete mesh geometry and the underlying smooth surface.

We first introduce the formulation of the rotational element. Figure 1(c) illustrates the cross-sectional profile of the compliant origami shell in the vicinity of the crease. Consider two adjacent triangular facets, denoted by $\mathcal{T}_1$ and $\mathcal{T}_2$, that share a common vertex located on the discrete crease line. In the discrete representation, geometric quantities are defined over triangular facets and, more specifically, along their edges. Within each facet $\mathcal{T}_1$ and $\mathcal{T}_2$, we connect the two vertices adjacent to the crease vertex, and the resulting line segment is referred to as the *mesh tangential* direction. This direction characterizes the discrete geometric orientation of each panel near the crease. To establish a consistent measure of the fold angle that reflects the underlying smooth geometry, we introduce a *virtual tangential* direction on each side of the crease. As illustrated in Figure 1(c), the virtual tangentials are constructed from the smooth cross-sectional profile of the origami shell. Specifically, they are defined as the tangent directions of the underlying smooth surface at the crease location. Let $\mathbf{t}_1^v$ and $\mathbf{t}_2^v$ denote the virtual tangential directions associated with $\mathcal{T}_1$ and $\mathcal{T}_2$, respectively. The virtual fold angle φ_v is defined as the signed dihedral angle between the corresponding orthogonal normal vectors, i.e.,

$$\varphi_v = \angle\big(\mathbf{n}_1^v, \mathbf{n}_2^v\big), \tag{S14}$$

where $\mathbf{n}_i^v$ are unit vectors orthogonal to $\mathbf{t}_i^v$. For clarity, tangential directions are illustrated in Figure 1(c), while the actual computation is performed using their orthogonal normals. The rotational element is modeled as a torsional spring distributed along the crease. The elastic energy density per unit length is given by $K_{\text{r}}\,(\varphi_v - \varphi_0)^2/2$, where K_{r} denotes the rotational stiffness and φ_0 is the rest angle. For a discrete crease segment shared by $\mathcal{T}_1$ and $\mathcal{T}_2$, the corresponding energy contribution is

$$U_{\text{rotation}} = \frac{1}{2} K_{\text{r}}\,(\varphi_v - \varphi_0)^2 \, \|\mathbf{e}_i\|, \tag{S15}$$

where $\|\mathbf{e}_i\|$ is the length of the common edge $\mathbf{e}_i$ between the two facets. For a discretized crease composed of multiple edge segments, the total rotational energy is obtained by summing the contributions over all segments. The additional bending element is introduced to characterize the bending of the facet pair adjacent to the crease. Since the rotational contribution is formulated in terms of the dihedral angle φ_v, it is natural to adopt a mid-edge discretization of the second fundamental form expressed in a dihedral-angle-based representation [5]. Similar to the definition of the virtual folding angle in Equation (S14), let $\mathbf{t}_1^m$ and $\mathbf{t}_2^m$ denote the mesh tangential directions extracted from the triangular facets. The dihedral angle of mesh tangential directions is defined through the orthogonal facet normals,

$$\varphi = \angle\big(\mathbf{n}_1^m, \mathbf{n}_2^m\big). \tag{S16}$$

In the dihedral-angle-based representation, the second fundamental form is expressed in terms of the dual edge direction $\mathbf{e}_i^*$, rather than the primal edge $\mathbf{e}_i$ adopted in Equation (S6). The dual edge is defined as the in-plane vector obtained by rotating $\mathbf{e}_i$ clockwise by 90°, i.e. $\mathbf{e}_i^* \perp \mathbf{e}_i$. Let $\mathcal{T}_1$ and $\mathcal{T}_2$ be two adjacent triangles sharing the common edge $\mathbf{e}_i$, and let φ denote the dihedral angle between their unit normals $\mathbf{n}_1$ and $\mathbf{n}_2$. The dihedral-angle-based representation of the discrete second fundamental form associated with edge $\mathbf{e}_i$ is written as

$$\mathbb{B}_i^{\text{dih}} = \frac{\sin(\varphi/2)}{a_i/2}\,\left(\bar{\mathbf{e}}_i^* \otimes \bar{\mathbf{e}}_i^*\right), \tag{S17}$$

where $\bar{\mathbf{e}}_i^* = \mathbf{e}_i^*/\|\mathbf{e}_i^*\|$ is the normalized dual edge. The geometric factor $a_i = 2A_{\mathcal{T}}/\|\mathbf{e}_i\|$ denotes the altitude of the triangle relative to the common edge, with $A_{\mathcal{T}}$ being the triangle area. Here, the discrete normal field is defined by averaging the adjacent face normals on the edge, so that the variation of the normal within each triangle corresponds to $\varphi/2$. The equivalence of the primal-edge (Equation (S6)) and dual-edge (Equation (S17)) representations has been rigorously established in [4]. The virtual fold angle φ_v defines a stress-free bending configuration. Evaluating the dihedral-based curvature at φ_v yields the reference second fundamental form $\bar{\mathbb{B}}_i^{\text{dih}} = \mathbb{B}_i^{\text{dih}}(\varphi_v)$. The curvature deviation associated with the crease is therefore expressed as

$$\mathbb{B}_i^{\text{dih}} - \bar{\mathbb{B}}_i^{\text{dih}} = \frac{\sin(\varphi/2 - \varphi_v/2)}{a_i/2}\,\left(\bar{\mathbf{e}}_i^* \otimes \bar{\mathbf{e}}_i^*\right). \tag{S18}$$

The difference $\varphi - \varphi_v$ therefore measures the deviation between the smooth geometric representation and the mesh-based geometry in the vicinity of the crease. This enables a direct geometric coupling between the bending measure and the crease rotation. Following the standard shell kinematics, this curvature difference induces an additional bending strain contribution

$$\boldsymbol{\kappa}_i^{\text{add}} = \bar{\mathbb{A}}_{ijk}^{-1}\left(\mathbb{B}_i^{\text{dih}} - \bar{\mathbb{B}}_i^{\text{dih}}\right), \tag{S19}$$

where $\bar{\mathbb{A}}_{ijk}$ denotes the reference first fundamental form. This additional bending strain is added to the conventional shell bending strain in Equation (S9) and contributes to the bending energy through Equation (S12). From a continuum mechanics perspective, the additional bending contribution can be interpreted as replacing the natural (free) boundary condition of the shell by a prescribed rotational constraint along the crease, induced by the virtual fold angle φ_v.

S1.4 Dynamic Formulation for Active Origami Systems

In addition to the internal elastic response arising from membrane, bending, and folding energies, the compliant origami robot is subjected to external environmental forces that are essential for reproducing realistic operational conditions. In this work, four types of external effects are incorporated into the simulation framework: gravitational loading $\mathbf{f}_{\text{g}}$, normal contact force $\mathbf{f}_{\text{contact}}^N$, tangential friction force $\mathbf{f}_{\text{contact}}^T$, and magnetic braking $\mathbf{f}_{\text{mag}}$. Within the proposed dynamic framework, all external contributions are assembled into a unified force vector $\mathbf{f}_{\text{ext}}$ entering the governing equations of motion, i.e.,

$$\mathbf{f}_{\text{ext}} = \mathbf{f}_{\text{g}} + \mathbf{f}_{\text{contact}}^N + \mathbf{f}_{\text{contact}}^T + \mathbf{f}_{\text{mag}}. \tag{S20}$$

It is worth noting that these environmental forces are applied only to physical vertices or panels and do not interact with the virtual crease elements. Consequently, the external force vector $\mathbf{f}_{\text{ext}}(t)$ contains zeros in its last N_2 entries, corresponding to the degrees of freedom associated with the crease variables.

Gravity. Gravity is modeled as a uniform body force acting on the mass of the origami sheet. Let ρ denote the surface mass density and h the thickness of the sheet. The gravitational acceleration vector is denoted by $\mathbf{g} \in \mathbb{R}^3$. At the continuum level, the gravitational force density per unit area reads $\mathbf{f}_g = \rho h \mathbf{g}$. After spatial discretization, the body force is converted into equivalent nodal forces consistent with the adopted mass formulation. Using a lumped mass representation, the gravitational force associated with vertex a becomes

$$\mathbf{f}_g^{(a)} = m_{(a)} \mathbf{g}, \tag{S21}$$

where $m_{(a)}$ is the lumped mass assigned to vertex a. The global gravitational contribution to the external force vector $\mathbf{f}_{\text{ext}}$ is therefore assembled as

$$\mathbf{f}_g = \left[m_{(1)}\mathbf{g}, \ldots, m_{(N_1)}\mathbf{g}, 0, \ldots, 0 \right]^{\mathsf{T}}. \tag{S22}$$

Normal Contact. Normal contact is introduced to prevent interpenetration between the origami sheet and a rigid obstacle, while enabling momentum exchange during impact events. Let d denote the signed gap distance between node a and the obstacle, and let $\tilde{d}$ be the prescribed contact threshold. The outward unit normal on the obstacle is denoted by $\mathbf{n} \in \mathbb{R}^3$. Contact is activated when $0 < d \leq \tilde{d}$. In practical implementation, penetration states with $d \leq 0$ are explicitly detected during the iterative solution process and handled through adaptive time step control to avoid invalid evaluations of the logarithmic term.

$$\Psi^N_{\text{contact}}(d) = k^N_{\text{contact}} \, (d - \tilde{d})^2 \ln\left(\frac{d}{\tilde{d}}\right), \qquad d \leq \tilde{d}, \tag{S23}$$

where k^N_{contact} is the normal contact stiffness parameter. And when $d > \tilde{d}$, $\Psi^N_{\text{contact}}(d) = 0$. The corresponding normal contact force is obtained from the potential gradient as

$$\mathbf{f}^N_{\text{contact}} = -\frac{\partial \Psi^N_{\text{contact}}}{\partial d} \mathbf{n}. \tag{S24}$$

For implicit time integration, the consistent tangent contribution along the normal direction reads

$$\mathbb{J}^N_{\text{contact}} = \frac{\partial^2 \Psi^N_{\text{contact}}}{\partial d^2} \left(\mathbf{n} \otimes \mathbf{n} \right). \tag{S25}$$

After assembly over all active nodes, the global normal contact contribution is incorporated into the external force vector $\mathbf{f}_{\text{ext}}$.

Tangential Contact. Tangential contact is introduced to account for frictional interaction along the contact interface. It is activated only when normal contact is active, i.e., when $d \leq \tilde{d}$. At the discrete level, the frictional response follows a regularized Coulomb-type law. Let $N = \left\| \mathbf{f}^N_{\text{contact}} \right\|$ denote the magnitude of the normal contact force, and let $\dot{\mathbf{v}}$ be the nodal velocity with scalar speed $v = \|\dot{\mathbf{v}}\|$. The friction coefficient is denoted by μ. The tangential contact force is defined in the direction of motion as

$$\mathbf{f}^T_{\text{contact}} = -\mu \, N \, f_{\text{frc}}(v) \, \frac{\dot{\mathbf{v}}}{\|\dot{\mathbf{v}}\|}, \qquad d \leq \tilde{d}. \tag{S26}$$

Here $f_{\text{frc}}(v)$ is a velocity-dependent regularization function introduced to ensure smooth behavior near zero velocity. A typical C^1-continuous choice reads

$$f_{\text{frc}}(v) = \begin{cases} 1, & v \geq \varepsilon_v, \\ -\left(\dfrac{v}{\varepsilon_v}\right)^2 + \dfrac{v}{\varepsilon_v}, & 0 \leq v < \varepsilon_v, \end{cases} \tag{S27}$$

where ε_v is a small regularization parameter controlling the transition to full sliding friction.

Magnetic Actuation. Magnetic actuation models the interaction between magnetized faces of the origami sheet and an externally applied magnetic field [8, 9]. Let $\mathbf{B}_{\text{ext}} \in \mathbb{R}^3$ denote a prescribed uniform external magnetic field. Each magnetic triangular element $\mathcal{T}$ is characterized by a remanent magnetic flux density $\mathbf{B}_{r,(\mathcal{T})}$ defined over its current configuration. The magnetic potential energy associated with element $\mathcal{T}$ is given by

$$\Psi_{\text{mag},(\mathcal{T})} = \int_{A_{(\mathcal{T})}} \mathbf{B}_{r,(\mathcal{T})} \cdot \mathbf{B}_{\text{ext}} \, \mathrm{d}A. \tag{S28}$$

Assuming a constant field over each element, this reduces to

$$\Psi_{\text{mag},(\mathcal{T})} = \big(\mathbf{B}_{r,(\mathcal{T})} \cdot \mathbf{B}_{\text{ext}}\big) \, A_{(\mathcal{T})}, \tag{S29}$$

where $A_{(\mathcal{T})}$ denotes the area of triangle. The total magnetic potential energy reads

$$\Psi_{\text{mag}} = \sum_{\mathcal{T}} \Psi_{\text{mag}}^{(\mathcal{T})}. \tag{S30}$$

The corresponding magnetic force and consistent tangent operator follow from the energy derivatives with respect to the nodal coordinates $\mathbf{v}$,

$$\mathbf{f}_{\text{mag}} = -\frac{\partial \Psi_{\text{mag}}}{\partial \mathbf{v}}, \qquad \mathbb{J}_{\text{mag}} = \frac{\partial \mathbf{f}_{\text{mag}}}{\partial \mathbf{v}}. \tag{S31}$$

After assembly over all magnetic faces, the global magnetic contribution is incorporated into the external force vector $\mathbf{f}_{\text{ext}}$.

S2 Ritz Approximation for the Folding Angle

To obtain an approximate analytical solution, we employ the Ritz method and assume a quadratic polynomial representation of the rotation field along the arc-length coordinate $s \in [0, W]$. Here, W denotes the length of one panel measured perpendicular to the crease. The rotation angle measured from the x-axis is denoted by $\theta(s)$. We assume

$$\theta(s) = \beta + as + bs^2 + c, \tag{S32}$$

where β represents the inclination angle of the straight configuration in the limit of vanishing rotational stiffness $K_{\mathrm{r}} = 0$. Define

$$\phi(s) = as + bs^2 + c, \tag{S33}$$

so that $\theta(s) = \beta + \phi(s)$. The coefficients a, b, and c are unknowns to be determined from energy minimization under constraint.

Bending Energy The bending energy of the panel is given by

$$U_b = D \int_0^W \left(\frac{d\theta}{ds} \right)^2 ds. \tag{S34}$$

Since

$$\frac{d\theta}{ds} = a + 2bs, \tag{S35}$$

integration yields

$$U_b = D \int_0^W (a + 2bs)^2 ds = D \left(a^2 W + 2abW^2 + \frac{4}{3} b^2 W^3 \right). \tag{S36}$$

Here,

$$D = \frac{Eh^3}{12} \tag{S37}$$

denotes the bending stiffness of the panel, where E is the Young's modulus and h is the panel thickness.

Crease Energy The rotation angle at the crease ($s = 0$) is

$$\theta(0) = \beta + c. \tag{S38}$$

The crease energy is therefore

$$U_c = \frac{1}{2} K_{\mathrm{r}} \left(\beta + c - \varphi_0 \right)^2, \tag{S39}$$

where φ_0 is the prescribed rest angle of the crease.

Geometric Constraint The horizontal projection length is prescribed by the imposed displacement, which leads to the constraint

$$x = \int_0^W \cos\theta(s)\, ds. \tag{S40}$$

Using a first-order linearization of the cosine function,

$$\cos\theta(s) \approx \cos\beta - \sin\beta\, \phi(s), \tag{S41}$$

the projected length becomes

$$x = \int_0^W \left(\cos\beta - \sin\beta\, \phi(s) \right) ds. \tag{S42}$$

Carrying out the integration yields

$$x = W\cos\beta - \sin\beta\left(\frac{aW^2}{2} + \frac{bW^3}{3} + cW\right). \tag{S43}$$

For any rotational stiffness, the projected length is required to remain equal to that of the rigid-folding configuration $\bar{x} = W\cos\beta$. Thus, the constraint reduces to

$$\frac{aW^2}{2} + \frac{bW^3}{3} + cW = 0. \tag{S44}$$

Augmented Functional Introducing a Lagrange multiplier λ, the augmented potential energy is defined as

$$U = U_b + U_c + \lambda\left(\frac{aW^2}{2} + \frac{bW^3}{3} + cW\right). \tag{S45}$$

Substituting the expressions above gives

$$\begin{aligned} U = {} & D\left(a^2W + 2abW^2 + \frac{4}{3}b^2W^3\right) \\ & + \frac{1}{2}K\left(\beta + c - \varphi_0\right)^2 \\ & + \lambda\left(\frac{aW^2}{2} + \frac{bW^3}{3} + cW\right). \end{aligned} \tag{S46}$$

Stationary Conditions The stationary solution satisfies

$$\frac{\partial U}{\partial a} = \frac{\partial U}{\partial b} = \frac{\partial U}{\partial c} = \frac{\partial U}{\partial \lambda} = 0, \tag{S47}$$

which leads to the algebraic system

$$\begin{cases} 2DW\,a + 2DW^2 b + \dfrac{\lambda W^2}{2} = 0, \\ 2DW^2 a + \dfrac{8}{3}DW^3 b + \dfrac{\lambda W^3}{3} = 0, \\ K(\beta + c - \varphi_0) + \lambda W = 0, \\ \dfrac{aW^2}{2} + \dfrac{bW^3}{3} + cW = 0. \end{cases} \tag{S48}$$

Solving the above linear system yields the coefficients a, b, c, and the Lagrange multiplier λ. In particular, the constant term is obtained explicitly as

$$c = \frac{WK_\mathrm{r}(\varphi_0 - \beta)}{WK_\mathrm{r} + 6D}. \tag{S49}$$

The folding angle at the crease is given by $\varphi = \theta(0)$, which leads to

$$\varphi = \beta + \frac{WK_\mathrm{r}(\varphi_0 - \beta)}{WK_\mathrm{r} + Eh^3/2}. \tag{S50}$$

S3 Finite element method (FEM)

Finite element simulations were performed in the commercial software **ABAQUS** to validate the proposed framework across different compliant origami configurations, including Miura structures and bending–crease coupled deformation cases. A unified FEM framework was adopted throughout all simulations, with differences arising primarily from the geometric configurations rather than the numerical implementation strategy. Each panel was modeled as an independent deformable shell part and discretized using four-node reduced-integration shell elements (S4R), with a thickness of $h = 0.01$ m and linearly elastic material properties ($E = 1.0 \times 10^6$ Pa). Panels were assembled via instance placement to form the complete tessellation, ensuring correct relative positioning. Mesh density was controlled by a fixed edge length parameter, with each edge divided into 20 elements, providing sufficient resolution to capture local deformations while maintaining computational efficiency. Special attention was given to the treatment of crease lines. Shared edges between adjacent panels were identified as crease lines, and torsional springs (Two-Point Spring) were applied along these edges to represent crease stiffness. Each torsional spring was defined directly on the finite element mesh nodes along the edge, with the spring stiffness evenly distributed across all nodes to capture the local rotational compliance accurately. External displacements were applied via reference points and coupled to the corresponding boundary nodes using equation constraints, ensuring that the overall motion of the assembly remained coherent while allowing rotation at the crease lines. The above procedure was automated using a Python script, and the code is available in an open-source repository `https://github.com/BhZhang-258/compliant_origami_robot`.

S4 Model Applicability and Computational Efficiency

This section provides additional details on the applicability and computational efficiency of the proposed DDG framework.

Model setup: The computational cost of the DDG model was compared with that of a shell-FEA benchmark. In the FEA model, the panels were modeled in Abaqus using S4R shell elements, and the creases were represented by rotational connector elements with prescribed elastic stiffness. The DDG and FEA models used consistent geometric, material, and crease-stiffness parameters. A key difference lies in the unknown variables of the nonlinear system. The S4R shell element uses both translational and rotational degrees of freedom at each node, whereas the present DDG formulation uses only nodal positions. Therefore, for a comparable number of nodes, the DDG model requires approximately half the number of equations of the corresponding shell-FEA model.

Computational cost: The total number of iterations is not directly comparable between the two solvers, because the DDG simulations used fixed loading increments, whereas Abaqus employs adaptive incrementation and optimized convergence controls. Therefore, the "Runtime / Iteration" column provides a more direct measure of the computational cost associated with solving one nonlinear equilibrium step. Tables S1 and S2 summarize the computational cost for the tensile and shear benchmarks. For the same mesh density, the DDG model contains fewer equations and shows a lower average runtime per nonlinear iteration; in the tested cases, this cost is reduced by about half or more compared with the shell-FEA model.

Table S1: Computational cost comparison for the tensile loading benchmark.

Method	Mesh density	Equations	Iterations	Runtime (ms)	Runtime / Iteration (ms)
FEA	10	1497	89	7000	78.65
DDG	10	637	145	1801	12.42
FEA	20	5377	90	8000	88.89
DDG	20	2477	135	6643	49.21

Table S2: Computational cost comparison for the shear loading benchmark.

Method	Mesh density	Equations	Iterations	Runtime (ms)	Runtime / Iteration (ms)
FEA	20	5377	269	23000	85.50
DDG	20	2477	306	13513	44.16

Although the two solvers use different increment strategies, the comparison between the tensile and shear benchmarks at the same mesh density still provides useful information on computational robustness. At a mesh density of 20, the deformation changes from the relatively simple tensile loading case in Table S1 to the more nonlinear shear loading case in Table S2. In this transition, the number of iterations in the Abaqus simulation increases from 90 to 269, nearly a threefold increase, which is consistent with its adaptive incrementation choosing more conservative steps to maintain convergence. By contrast, the DDG simulation remains stable under fixed loading increments, and the number of iterations increases from 135 to 306, approximately a twofold increase. This comparison suggests that the DDG formulation retains favorable numerical robustness in the tested strongly nonlinear origami deformation problem. This robustness may be related to its reduced set of field variables and the lower continuity requirement imposed on the deformation field.

Mesh convergence: Figure S1 further evaluates the mesh-refinement behavior of the DDG model. The error decreases steadily as the mesh is refined, indicating that the DDG formulation can recover increasingly resolved shell-like deformation. The observed convergence trend is comparable to that of the low-order shell-FEA benchmark. At the same mesh density, the absolute error of DDG can be larger than

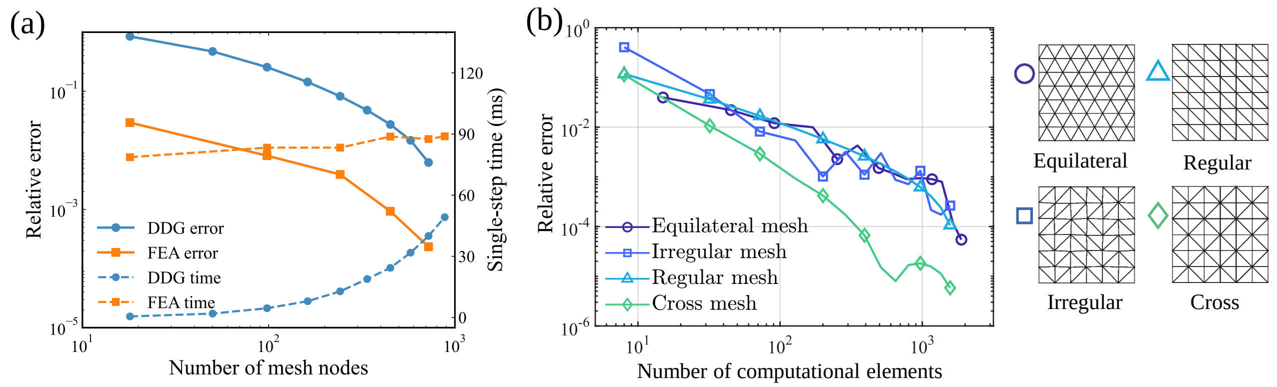

Figure S1: Mesh convergence and discretization dependence of the DDG model. (a) Error convergence of DDG and shell FEA under mesh refinement. The DDG solution converges steadily with mesh refinement and shows a convergence trend comparable to the low-order shell-FEA benchmark, although the absolute error can be larger at the same mesh density because of the reduced nodal variables and lower-order geometric representation. (b) DDG convergence behavior under different triangulation patterns. Once a sufficient mesh density is used, the main deformation response is not strongly dependent on a specific edge discretization or crease segmentation.

that of FEA, which is expected because the DDG model uses a reduced set of variables and a lower-order discrete geometric representation.

Model applicability: The mesh density should be selected according to the deformation mode of interest. For crease-dominated rigid-like folding, a coarse discretization can already capture the primary folding motion. When panel bending becomes important, at least several elements are required across each panel to represent the relevant curvature mode. For highly compliant origami involving coupled stretching, bending, twisting, and nonuniform curvature, a finer mesh is needed to approximate continuum shell deformation. This tunable resolution is central to the proposed framework: the same DDG formulation can be used with coarse meshes for rapid design exploration, or with refined meshes for more accurate simulations of complex compliant deformation.

S5 Experimental Comparison of the Compliant crawling Robot Simulation

To examine the predictive capability of the proposed DDG framework for compliant origami robots, we compared the simulation results with the experimental data reported in Ref. [10]. The comparison includes the mechanical response of a compliant Kresling origami actuation unit and the magnetic-field-induced contraction of the full robot.

buckling phenomenon The compliant Kresling origami actuation unit used for comparison is the one shown in Figure. 7a–d. In the DDG model, the material and geometric parameters are chosen consistently with the reported experimental system. The experimental data are from Ref. [10]. Figures S2a and S2b compare the simulated and measured force–displacement responses of the actuation unit. The DDG simulation captures the two-stage mechanical response of the structure. Before buckling, the force–displacement curve shows a relatively large slope, indicating a high initial stiffness. After buckling, the slope becomes much smaller. This transition is consistent with the mechanical characteristics of the Kresling structure discussed in Figure. 6 and 7. Specifically, the intrinsic folding mode of the Kresling structure relies on in-plane stretching of the panels to accommodate the folding deformation, whereas the compliant Kresling structure can release this constraint by switching the dominant panel deformation from stretching to bending.

The energy landscape in Figure. 7c further supports this interpretation. At the early stage of folding, the stretching energy dominates the total elastic energy, indicating that the panels mainly undergo in-plane deformation. After the critical point, the bending energy emerges and becomes dominant, suggesting a transition from stretching-dominated deformation to bending-dominated deformation. This energetic transition is reflected in the force–displacement responses in Figure. S2a and S2b: the structure exhibits a large effective stiffness before the critical point, followed by a much softer response after buckling.

The energy response is further compared in Figure. S2c. The DDG result follows the experimentally observed increase in stored elastic energy during loading, suggesting that the framework can capture not only the deformation mode but also the associated energetic trend.

Analysis of discrepancies It should be noted that thin-shell origami structures are often sensitive to imperfections. As a result, the mechanical response of the compliant Kresling unit may change with repeated loading. Repeated actuation can introduce initial imperfections associated with plastic deformation, residual curvature, crease damage, or material-history effects, which may shift the apparent buckling point and reduce the slope of the pre-buckling response, as shown in Figure. S2b.

Moreover, the lack of experimentally measured parameters in the reported data limits the accuracy of direct quantitative comparison between the DDG model and the tested soft origami robot. A more precise match would require dedicated calibration of the effective crease stiffness and other structure-dependent factors, such as local plasticity, residual deformation, and fabrication-induced imperfections. Nevertheless, Figure S2a and S2b show that the DDG simulation agrees reasonably well with the measured responses and captures the dominant buckling-induced stiffness transition.

Magnetic actuation For the full robot, we simulated the deformation induced by magnetic actuation and compared it with the experimentally observed actuation behavior, as shown in Figure. S2d. The DDG simulation captures the magnetic-field-induced contraction curve, indicating its potential for guiding robot-level actuation and control design. A slight discrepancy appears in the latter part of the curve, where the contraction ratio becomes relatively large. The experimental response gradually approaches a plateau as the magnetic field increases, whereas the DDG result continues to increase more smoothly. This discrepancy is likely related to the additional constraints induced by the finite thickness of the experimental panels, which limit the maximum compression of the structure to approximately 30%. Overall, the comparison indicates that the proposed DDG framework can reproduce the dominant deformation mechanisms, energetic trends, and actuation response of compliant origami robots, while retaining a

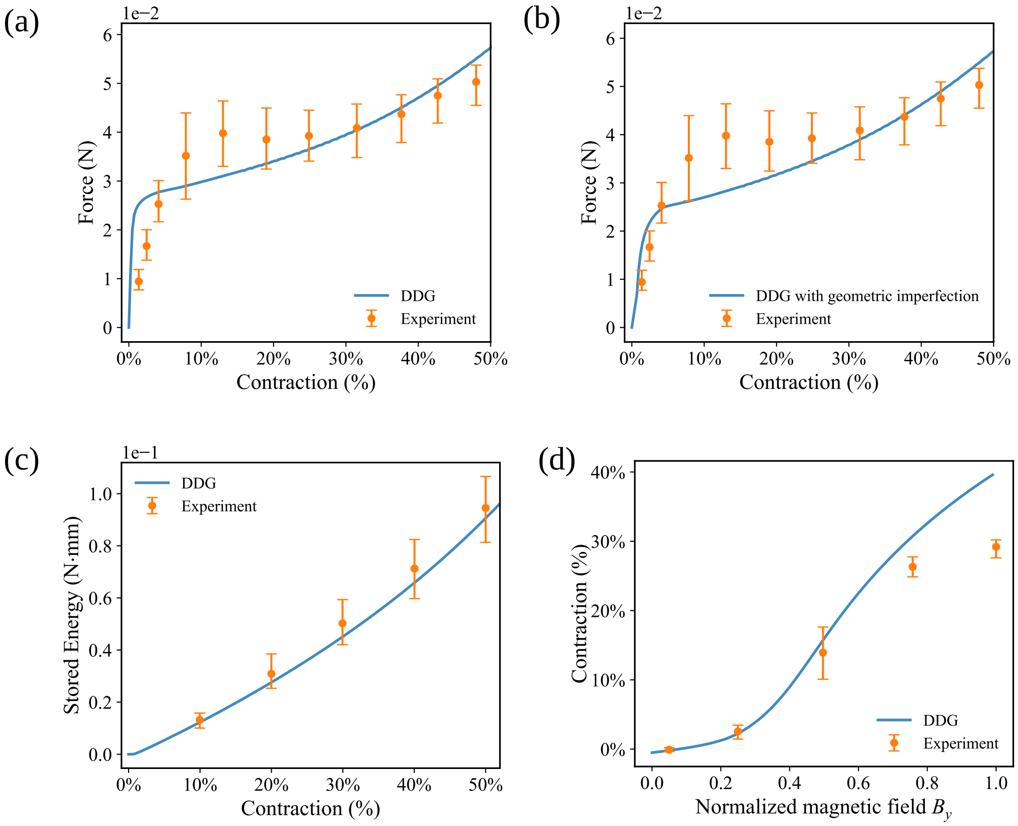

Figure S2: Experimental comparison of the DDG simulation for the compliant crawling origami robot. (a,b) Force–displacement responses of the compliant Kresling origami actuation unit (shown in Figure. 7a-d), comparing DDG simulation with experimental measurements. (c) Stored elastic energy during loading. (d) Magnetic-field-induced contraction of the full crawling robot. Experimental data are adapted from Ref. [10].

reduced and design-oriented modeling form.

S6 Video

We provide a video as supporting Information to show the dynamic behaviors of the flexible origami system.

References

[1] B. Zhang, B. Wang, H. Ouyang, Z. Wu, H. Bi, M. Liu, W. Huang, Supplementary material code, `https://github.com/BhZhang-258/compliant_origami_robot`, **2026**.

[2] W. Huang, Z. Hao, J. Li, D. Tong, K. Guo, Y. Zhang, H. Gao, K. J. Hsia, M. Liu, *Applied Mechanics Reviews* **2025**, 1–88.

[3] D. Tong, A. Choi, J. Wang, W. Huang, Z. Chen, J. Li, X. Huang, M. Liu, H. Gao, K. J. Hsia, *Extreme Mechanics Letters* **2026**, *82* 102430.

[4] W. Clarisse, Ph.D. thesis, University of Göttingen, **2012**.

[5] E. Grinspun, M. Desbrun, K. Polthier, P. Schröder, A. Stern, *ACM Siggraph Course* **2006**, *7*, 1.

[6] H.-Y. Chen, A. Sastry, W. M. Van Rees, E. Vouga, *ACM Transactions on Graphics (TOG)* **2018**, *37*, 4 1.

[7] S. Armon, E. Efrati, R. Kupferman, E. Sharon, *Science* **2011**, *333*, 6050 1726.

[8] J. Sim, L. Lu, R. R. Zhao, *Advanced Materials* **2026**, *38*, 11 e21268.

[9] L. Wang, Y. Chang, S. Wu, R. R. Zhao, W. Chen, *Nature Communications* **2023**, *14*, 1 8516.

[10] Q. Ze, S. Wu, J. Nishikawa, J. Dai, Y. Sun, S. Leanza, C. Zemelka, L. S. Novelino, G. H. Paulino, R. R. Zhao, *Science Advances* **2022**, *8*, 13 eabm7834.
